# Location Privacy Protection in Distributed IoT Environments Based on Dynamic Sensor Node Clustering

**DOI:** 10.3390/s19133022

**Published:** 2019-07-09

**Authors:** Konstantinos Dimitriou, Ioanna Roussaki

**Affiliations:** 1 School of Electrical and Computer Engineering, National Technical University of Athens and Greece, 15773 Athens, Greece; 2 Institute of Communication and Computer Systems, 10682 Athens, Greece

**Keywords:** internet of things (IoT), data-centric sensor networks (DCSNs), wireless sensor networks, location privacy protection, dynamic node clustering, FIT-IoT lab

## Abstract

One of the most significant challenges in Internet of Things (IoT) environments is the protection of privacy. Failing to guarantee the privacy of sensitive data collected and shared over IoT infrastructures is a critical barrier that delays the wide penetration of IoT technologies in several user-centric application domains. Location information is the most common dynamic information monitored and lies among the most sensitive ones from a privacy perspective. This article introduces a novel mechanism that aims to protect the privacy of location information across Data Centric Sensor Networks (DCSNs) that monitor the location of mobile objects in IoT systems. The respective data dissemination protocols proposed enhance the security of DCSNs rendering them less vulnerable to intruders interested in obtaining the location information monitored. In this respect, a dynamic clustering algorithm is that clusters the DCSN nodes not only based on the network topology, but also considering the current location of the objects monitored. The proposed techniques do not focus on the prevention of attacks, but on enhancing the privacy of sensitive location information once IoT nodes have been compromised. They have been extensively assessed via series of experiments conducted over the IoT infrastructure of FIT IoT-LAB and the respective evaluation results indicate that the dynamic clustering algorithm proposed significantly outperforms existing solutions focusing on enhancing the privacy of location information in IoT.

## 1. Introduction

The Internet of Things (IoT) [1] is an emerging technology paradigm that is gaining momentum, constantly attracts the attention of scientific and industrial communities and which has penetrated the market in a plethora of domains. It has the potential to greatly impact people’s lives and to enhance various aspects in several industries, such as health, transportation, energy, manufacturing, smart cities, agrifood, etc. The IoT connected devices installed worldwide are expected to rapidly increase in the coming years and exceed 75 billion by the year 2025 (https://www.statista.com/statistics/471264/iot-number-of-connected-devices-worldwide/). Many manufacturers are currently investing in the establishment of IοT infrastructures in order to improve monitoring of their resources, facilitate their production processes and enhance the distribution of their products, aiming for cost reduction and quality improvements. Moreover, data collected by IoT infrastructures are used by numerous providers to increase the value of the services or applications they offer. In this respect, a wealth of IoT-based applications is using location information tailoring their features to the location of the user or object they concern. However, there are various barriers that significantly delay the further promotion and evolution of the IoT vision, such as matters regarding privacy, security and user acceptance. Having been integrated in our everyday lives and being able to collect and distribute large volumes of (often sensitive) data, IoT has attracted the interest of malicious intruders that frequently attack IoT infrastructures to gain access to potentially valuable information.

Wireless sensor networks [1] are a critical component of the IoT and have been extensively used for various purposes in IoT environments, such as the surveillance of specific areas. Wireless sensor networks comprise spatially located sensor-equipped devices that are able to track their surroundings. These sensors can monitor the physical conditions of the environment or mobile objects that enter the network range. The collected data are aggregated and organized at a central location. Their widespread usage has led to the collection of large volumes of sensed data, as well as requirements for enormous amounts of energy. Thus, a new generation of data-centric sensor networks (DCSNs) [2,3] has been developed. In these networks, the data originating from the detection nodes (usually sensor nodes with limited resources) are stored in other nodes that are responsible for maintaining the respective data and that can engage the required resources to support this. Most of the times, the sensor nodes are scattered in random positions and that poses some difficulties when it comes to receive some queries. This situation can impede the communication between legitimate users and a specific set of sensor nodes or lead to major power consumption. That is exactly the reason why the DCSNs use data centric routing. The queries are sent to specific regions of sensor nodes where the data is aggregated. In this way, the latency of messages and the power consumption can be reduced by this aggregation of data. Even though these networks are quite efficient with respect to data synchronization and energy consumption matters, they are vulnerable to attacks by various aspiring intruders. Their goal is either to obtain access over the data of a storage node or to cease its operation so that the data it maintains are not available to the rest of the IoT infrastructure. In order to mitigate such attacks, various cryptographic methods have been proposed to ensure the privacy and confidentiality of IoT data. However, these techniques are not sufficient to guarantee full privacy protection, while they introduce considerable complexity and increase the required computational resources [4,5]. Therefore, a number of other techniques that are not based on cryptographic methods have been developed that require low resources and aim to enhance the level of privacy ensured.

This article examines a privacy issue regarding the monitoring functionality of DCSNs. We deploy a network of sensors which are able to detect each mobile node that passes through their monitoring range. The detection range of sensor nodes constitutes a circle of specific radius whose centroid is the position of sensor node itself. The localization of the position of the mobile node is achieved through the intersection of their detection range. The network comprises of a specific number of storage nodes which is responsible for calculating the intersection areas when receiving the corresponding messages from sensor nodes. The narrower the intersection area is, the more precise we can locate the mobile object. However, an emerging privacy issue of such networks is when an intruder manages to compromise a storage node and gains access to its data.

To address the case where the area of the intersection that storage node maintains is narrow and the location of the mobile object can be precisely extracted, we introduced an innovative mechanism that aims to protect the privacy of location information across DCSNs that monitor the location of mobile objects in IoT infrastructures. In this respect, two clustering algorithms are introduced that do not focus on the prevention of attacks, but on enhancing the privacy of sensitive location information once IoT nodes have been compromised. The algorithm introduced in this paper is designed to solve “real-world” problems in real systems [4]. The orientation of our research is the preservation of users’ data and privacy rather than avoiding the compromise of the nodes themselves. If an intruder managed to gain access to some storage nodes, it would be difficult for him to infer the precise location of the user or acquire personal data. Popular applications of WSNs such as car tracking or baby monitoring or even wild animal tracking can be exploited by malicious users to discover the locations of such targets. This second layer of privacy that this innovative algorithm achieves can ensure a higher level of security regarding user’s valuable data.

The rest of this article is structured as follows: in Section 2, the related state of the art work is reviewed and elaborated upon. In Section 3, the IoT sensor network model employed is described in detail. Section 4 presents the location privacy protection problem to be addressed and introduces the problem concepts, setting and formal statement, as well as the respective evaluation metrics to be used. Section 5 introduces the proposed solution and presents the dynamic clustering algorithm designed and implemented to address the respective problem. Section 6 elaborates on the series of experiments conducted to assess the proposed mechanism and presents the respective evaluation findings. Finally, in Section 7, conclusions are drawn and future plans are presented.

## 2. Related Work

### 2.1. Privacy of Location in IoT Environments

There are several emerging problems in the context of IoT that have attracted significant attention across the research community. In this respect, privacy preservation and security protection of IoT devices and users have become one of the most challenging topics over the last decades. Lopez et al. [6] have studied the most common privacy issues and the most prominent countermeasures regarding wireless sensor networks in IoT. They have conducted extensive research in various fields, such as user privacy, content privacy and context privacy. More specifically, location information leakage can lead to critical data privacy problems. In this framework, Chen et al. [7] have evaluated threats and concerns for both Global Navigation Satellite Systems (GNSS) and non-GNSS solutions. Their research focuses on cryptographic techniques regarding Location-based Services (LBS) and state-of-the-art regulations. However, the existing techniques for LBSs in IoT cannot be implemented without inducing heavy processing overheads. Lin et al. [8] have conducted a detailed survey studying the threats and existing solutions for ensuring privacy in Smart Home applications and they propose gateway architectures as the most prominent and appropriate way to guarantee this. Two important factors are identified in [8] as the key elements to render a system more robust and secure: the automatic installation and configuration. The former is the auto-configuration of IoT devices and systems that will improve the level of privacy. The latter is the automatic update of system software and firmware to fix unpatched bugs and vulnerabilities.

The provision of location-based services (LBSs) is frequently supported by suitable location monitoring IoT infrastructures. The exposure of trajectory information and location data in such services introduces critical privacy concerns. Peng et al. [9] have conducted extensive research to address issues concerning continuous queries from which adversaries can extract private location information. Two techniques are proposed in this paper to address this: the multi-hop caching-aware cloaking algorithm (MCC) and the collaborative privacy-preserving querying algorithm (CPPQ). In MCC, users exchange information, regarding their location, with multi-hop peers. CPPQ algorithm seeks for information that has been stored in local caches and issues a fake query to confuse the location service provider and the adversary. However, the proposed scheme cannot be implemented when collaborative users are untrustworthy. Liao et al. [10] studied the problem of location and trajectory privacy preservation in 5G-based vehicle social networks (VSNs). They proposed the Dynamic Group and Division Algorithm (DGD) that exploits the Group Generating Protocol and Pseudonym Exchanging Protocol. The vehicles of the network are dynamically divided into group regions based on their location and via the Pseudonym Exchanging Protocol they employ, they exchange pseudonyms instead of their real group identity. This approach increases the difficulty for the attackers to deduce the real identity of the car within a group region and track its trajectory. However, this research is based on simulations and is not tested in real-world scenarios and experiments over existing IoT infrastructures.

As already mentioned, major privacy preservation problems are caused by mobile applications and LBSs in IoT environments that use the current location of the user in order to adapt the provided service accordingly. Intruders exploit such services and launch attacks based on continuous user queries to infer their location. Ma et al. [11] introduced the EPPING algorithm to address such issues employing an anonymous trusted LBS server to process KNN queries. Users are not obliged to transmit their precise location but a cyclic region. EPPING performs depth first search algorithm to find the KNN points of the region for specific confidence level thresholds. The drawbacks of the proposed solution are the higher network traffic and the fact that it is hard to find a trusted anonymous server in real-world scenarios. Hashem et al. [12] have also studied privacy preservation problems regarding location based services that expose users’ exact location. They proposed a novel framework based on k group nearest neighbor (kGNN) queries. The kGNN queries return a place that minimizes the aggregated distance from a distributed group of users. This novel framework employs a location service provider (LSP) that returns a set of candidate answers that includes the actual kGNN. The selection of the appropriate place among the set of candidate answers is determined via the final pruning private filter (FPPF) or incremental pruning private filter (IPPF), which do not expose private data to attackers or third party services. However, the amount of candidate answers needs to be further reduced, while privacy is not sufficiently preserved in road networks.

With the advent of IoT services and devices, the integration of location-based services was inevitable. Su et al. [13] proposed location-label based algorithm (LLB) to tackle the problem of protecting the users’ identity when their location is exactly the same or similar in IoT environments. The LLB algorithm attaches labels to users and comprises of 3 steps. The first is the request aggregation protocol where user aggregates other users’ labels, the second is pseudo-ID exchanging protocol in which users exchange their PIDs when they are in sensitive locations and third is the implementation of improved-PALM protocol that Niu et al. [14] introduced. Additionally, Sun et al. [15] studied the problem of untrusted or malicious LBSs that can expose personal data or affect users’ privacy. They introduced the Dummy Location Privacy-Preserving (DLP) algorithm which chooses the optimal dummy locations to prevent the attacker from extracting real users’ locations. Finally, Gonzalez-Manzano et al. [16] designed PagIoT, a privacy-preserving aggregation protocol that contributes to the aggregation of data and the correlation between the values. However, the aforementioned protocols have not been tested to real-world operational environments that differ greatly from simulation environments and strong limitations have been assumed to evaluate these.

### 2.2. User Anonymity and Authentication Privacy in IoT Systems

The preservation of user anonymity when accessing decentralized location based services such as IoT environments constitutes a crucial privacy issue. Intruders that have access to IoT networks and devices should not be able to extract user’s identity so as to breach personal data. Bettini et al. [17] proposed a general framework based on already existing approaches. This general framework intends to classify the attackers into groups based on the level of knowledge that is exposed to them in such networks. This research does not provide preventive solutions to possible attacks for LBS servers but is intended to model different scenarios aiming to partition them into classes. A proposed extension of this framework constitutes the modeling of attacks with multiple requests. However, the conservation of anonymity is not always feasible and not all proposed methods can fully guarantee users’ privacy. Lin et al. [18] examined the fully decentralized anonymous authentication protocol that Alcaide et al. [19] proposed and found that it can be insecure under certain circumstances. In specific, attackers that impersonate legitimate users can steal personal data and breach privacy. Elkhodr et al. [20] introduced a context-aware adaptive approach to enhance privacy when accessing location-based protocol. This approach utilizes a Dynamic Location Disclosure Agent (DLDA) to protect users’ location. DLDA constitutes a system that takes as input the actual location of the user and performs Dynamic Disclosure-Control Method (DCCM) to obfuscate it. However, this technique has not been tested in a fully decentralized environment such as IoT systems. Another drawback of this method is that the processing power of ΙοΤ devices is limited and that could impose risks to the demanding computations of the attached agents.

### 2.3. Storage System Architectures in IoT

A very important part of a wireless detection network [4,21] is its storage nodes and, in general, the system according to which the data is stored. A very important study on this issue is Safestore, which is a distributed storage system designed to maintain long-term data regardless of any network failure, human error or malicious attack [22]. Thus, innovative 2-level architecture is proposed, consisting of a local server that acts as intermediate storage and a remote system with multiple storage providers. Advantages of this architecture are the efficient dissemination of data between different providers as well as the effective edge-to-edge control of data for possible loss of data. It offers storage at cost, performance, and availability compared to conventional storage systems. However, this architecture is not resistant to resource consumption attack as an intruder could send to the server a large amount of data leading to denial of service attack.

Nodes and storage systems on detection networks should keep the data and access it regardless of the problems that may arise either on the server side or on the client side. That’s why PASIS architecture [23] was developed to solve server problems, such as server connections, coding data with threshold patterns, and making servers more secure. The self-securing systems proposed in this study solve client-side problems by monitoring access to and processing data. It uses p-m-n threshold schemes where data breaks into n parts, any m of them can generate the original data, and p does not give any information. The PASIS architecture consists of a distributed storage system and some client-side agents to the user side that collect the parts of the data shared in the storage system and combine them using the threshold schemes. However, this architecture suffers from data recovery problems as it can take a long time for the data to be restored from backup.

### 2.4. Traffic Monitoring and Analysis of Privacy Issues in IoT Environments

Monitoring and analyzing messages from a wireless network by aspiring intruders is a very important problem as it can lead to a variety of attacks. This problem becomes even more pronounced especially when the potential intruder is able to monitor the entire network. A study conducted on this topic introduced the concept of false traffic within the network with two different methods of proxy-based filtering scheme (PFS) and tree-based filtering scheme (TFS) [24], which is difficult and expensive to be implemented. In the first method, some of the detection nodes are selected as proxy nodes that have the ability to filter the packets. Messages on the network are sent at intervals that follow the exponential distribution so that false traffic does not separate from actual events. When a fake package reaches a proxy node then it is discarded. Instead, when a real event arrives, it is re-encrypted and sent to the base station for storage. In the event that no real event comes, then an encrypted fake message is sent. The second method is to solve the problem of a large number of proxy nodes by organizing them in a tree form. So the message is filtered into multiple proxy nodes and traffic is reduced. Of course, this method increases the time that a real event is reaching the base station. 

An equally important problem that can be generated by monitoring the traffic of a network from a node to a node is the export of the source location. A large field of research is the communication protocols of the nodes within the network and many have been created for security purposes such as phantom routing [25]. This study analyzes already existing protocols such as flooding routing, in which a node that wishes to transmit a message promotes it to all its neighbors and they in turn to theirs, creating a flood of messages on the network. It also examines single path routing in which nodes forward messages to only a neighbor who decides through various techniques and routing with fake sources to which various fake sources are generated that generate fake network traffic to confuse the attacker. These techniques are either easily perceived by the attacker or consuming extra energy. Thus, a new technique is proposed for phantom routing, in which a phantom source is introduced. There are two steps: (1) the phase of the random walk to the ghost source and (2) the implementation of flooding/single-path routing for the transmission of the message to the destination. However, this technique has not yet been fully tested to more complex adversarial models.

All of the above techniques may be quite vulnerable, especially when the intruder can monitor and know the entire network. To avoid cases of information leakage and site exports, under these circumstances, two techniques, periodic collection and source simulation [26] have been proposed. In the first technique, each node independently, at regular intervals, sends data at a reasonable frequency whether it is real events or false messages. However, despite the security it gives to the network, it consumes large amounts of energy in real-time applications. In the second technique, a set of fake mobile objects are entered and assimilated into the network as if they were true. In this way, an attacker cannot distinguish true targets from false. However, this technique does not deal with the fact that an eavesdropper is able to compromise nodes of the network and acquire sensitive data.

Another major issue faced by wireless detection networks is the traffic monitoring attacks by aspiring intruders. Such networks consist of sensing sensors and base stations that collect the data. Two types of attacks targeting these stations have been studied, as well as various methods to deal with them [27]. The first family of attacks involves isolating and blocking communication between the base station and the other nodes, which can be addressed by establishing multiple paths on multiple base stations and using techniques such as one-way hash chains and echo-back algorithms. The base stations generate a REQ message that floods the network and is received by the nearest nodes, which in turn forward it to their child nodes, thus creating multiple paths to the base stations. To prevent an attacker from creating a malicious REQ message, the one-way hash chain technique can be employed. As far as the echo-back algorithm technique is concerned, whenever a detection node receives one REQ from another, it simultaneously sends an echo message to the second waiting for its response. In this way, he can safely recognize his neighbors. The second family of attacks involves analyzing traffic to a base node to extract its location. Techniques to deal with such attacks are hop by hop encryption/decryption and sending rate control. In the first method, each node also has a cluster key to be able to encrypt the message it sends. In the second technique, the rate of sending of each node is controlled. However, it does not constitute a scalable solution as there are overheads as the network is constantly getting larger.

The need for efficient data transmission and secure access to network data have led to the creation of data-centric sensor networks (DCSN). However, various security problems created by aspiring intruders led to the creation of a new generation of pDCS networks, which offer different levels of security, based on different cryptographic keys [28]. In addition to this study, two techniques for query optimization, Euclidean Steiner Tree (EST) and Keyed Bloom Filter (KBF) are proposed. With regard to query optimization in the first technique, EST, there are some cells called Steiner cells different from the storage cells and are organized in the EST tree format with the top of the mobile sink that generates the query. This node advances the message to his children, which build EST subtrees and return the message until it reaches the storage nodes. In the second technique, KBF, we have a set of *S* = *s*_1_, *s*_2_, ..., *s_n_*, *k* hash functions and a string of m bits initialized to 0. For each *s* ∈ *S*, we put it as an input to each of *k* functions and obtain the values *h_i_*(*s*) (1 ≤ *i* ≤ *k*). The bits corresponding to these values are set to value 1. In addition, the cell keys are used to encrypt the id of the storage nodes before they enter the algorithm. However, this work is based on simulation results and not real world experiments.

### 2.5. Integration of IoT Devices with Cloud Systems

Cloud computing has gained great popularity over the last decades, especially for IoT systems [29,30] due to the cost reduction, data security and scalability that it offers. However, aside from the appealing features that it provides, it can cause a plethora of data privacy problems, especially when it comes to outsourcing sensitive data to third-party cloud environments. The recommended strategy is to transfer data in encrypted format yet this impedes searching function. Fu et al. [31] proposed a similarity search method for encrypted documents based on simhash. In this new scheme, the documents are transformed into fingerprints with simhash and hamming distance is used to compare them. Furthermore, a tree-based approach is utilized so as to reduce the searching time.

## 3. IoT Sensor Network Model

In this section, the IoT sensor network model used in the framework of this study is presented. It is based on the model introduced by Miao Xu et al. [2] and is built around the concepts discussed hereafter.

*Sensor Nodes*: The network model consists of sensing nodes that are able to detect the presence of moving objects within their vicinity. These are arranged in a flat space (2-dimensional problem) and are denoted by *x*_1_, *x*_2_, ..., *x_n_*. All nodes are identical as they have the same detection radius *r_s_*. They are responsible for monitoring their surrounding environment, detecting mobile objects and sending messages to a storage node, once an object is detected. The detection process is as follows. The nodes can detect the moving target if it moves at a distance within their detection radius. The information about the message being sent only indicates that the mobile object is currently located within their detection range. More specifically, the information sent from sensor nodes to storage nodes is when the mobile object is detected encapsulates only a timestamp, an ID (uniquely identifying the mobile object) and a Boolean parameter (set to TRUE if the mobile object is detected or to FALSE when the mobile object is no longer detected). Nodes can store limited information due to a lack of memory and they only know the relative positions of their neighbors. The sensors are used to localize the mobile target without revealing its precise location. They do not support exact localization of objects or distance estimation.

*Storage Nodes*: The network consists of storage nodes that are arranged in a flat space and are denoted by *y*_1_, *y*_2_, ..., *y_m_*. It is assumed that the population m of storage nodes is much lower than the population n of sensor nodes. The lower population m is, the strongest the location privacy risks are. The storage nodes have sufficient memory, large battery stocks and are responsible for receiving the messages sent by the sensor nodes and processing the respective information properly. For this reason, despite the cryptographic methods used in the network, they should have access to the non-encrypted text. Finally, they have the ability to do some kind of packet filtering to ensure protection from malicious attacks.

*I-state*: At time *t* the *I*-state represents the set of all potential target object positions consistent with the detection messages with timestamps prior to time t. The size of the area corresponding to an *I*-state thus quantifies the level of uncertainty concerning the location of the target object. A larger value for this area suggests that the target object can be located anywhere inside a larger area, corresponding to a higher level of uncertainty regarding its location. Each storage node *y_j_* maintains its own *I*-state, denoted by *IS_j_*(*t*), which corresponds to potential location area *LA_j_*(*t*) of the target mobile object and is updated constantly, also based on the detection messages it receives. Of course, in case storage node *j* does not store any information regarding the location of the target object at time *t*, then *LA_j_*(*t*) is equal to the entire area monitored by the IoT infrastructure deployed.

*Global Node*: This is the only storage node that always receives the object detection messages of all sensor nodes that detect the target mobile object. The Global node maintains its own *I*-state, denoted by IS^*^(*t*), which aggregates the *I*-states of all storage nodes $IS^{*}\left(t\right)=\underset{j}{\cup }IS_{j}\left(t\right)$ and corresponds to the intersection of their potential location areas $LA^{*}\left(t\right)=\underset{j}{\cap }LA_{j}\left(t\right)$. It is equipped with special hardware to provide enhanced protection. It is assumed that the Global node is trustworthy and cannot be tampered with by an aspiring intruder.

*Message processing*: The messages sent by sensor nodes are processed by the storage nodes upon their reception. Each storage node maintains its own *I*-state which is the set of possible positions of the target node based on the messages that were received prior to a specific time. The calculation of the *I*-state is based on the following procedure:

- When no message is received within an interval *t*_2_ − *t*_1_ sec, then *I*-state of time *t*_2_ is computed from the one of *t*_1_ by performing a Minkowski sum [32] between the *I*-state of time *t*_1_ and a circle of radius (*t*_2_ − *t*_1_)∙*v*_max_, where *v*_max_ is the maximum velocity that the target object can reach. Intuitively, the *I*-state expands itself tο include the possibility that the target object may have moved outside the detection range the respective storage node may cover.
- When a storage node receives a message, its *I*-state is updated to the intersection of the previous *I*-state with the coverage area of the sensor node posting the message. Thus, the storage node always takes into account the region where the detection message is sent from.

**Figure 1:**
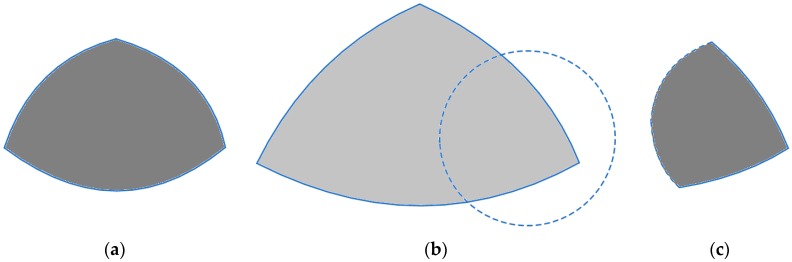
Computing the potential location area of the *I*-state: (**a**) LA of an initial information state; (**b**) expansion to account for the passage of time and intersection with range of the sensor node posting the detection message; (**c**) the resulting LA of the updated *I*-state [2].

Figure 1 briefly illustrates the proposed procedure of message processing:

All network nodes beyond the global node can be tampered with and considered unreliable. The intruder can violate at least 1 node of the network. In this research we focus on the scenario of compromising one storage node that is of higher interest due to the sensitive data it maintains. Additionally, a trajectory of the target object is deemed to be valid if at least one sensor node of the network is able to detect it. The intruders do not have a global view of the network, but are only aware of a partial view that has been deduced based on the information they have intercepted from the storage node they compromised.

The network introduced above is the basis of our research where our innovative algorithm is applied. It constitutes a DCSN that comprises of multiple types of nodes such as sensor nodes, storage nodes, Global node and mobile. The data collected by sensor nodes are aggregated to storage nodes.

## 4. Problem Concepts and Definition

### 4.1. Problem Concepts and Setting

Location detection refers to the ability of the IoT system to identify the location of the target object that moves within its infrastructure. However, as already stated, the aggregation of location data imposes a plethora of privacy problems that can be divided into two main categories. The former is the unauthorized access to personal data and the latter is the granularity of location exposure. Depending on what is the target object and how dense the sensing network is, the granularity of detection can significantly differ. Revealing house addresses, military base station locations, etc., is unacceptable and is treated as major privacy breach [4]. The positions of the sensor nodes on the flat space, the distance between them, the sensing range and the employed data aggregation policies are some of the parameters that should be considered to effectively ensure the protection of location information. The research work presented herewith focuses on the second category and the basic problem addressed is the definition of a location data transmission protocol between the detection sensor nodes and the appointed storage nodes. The main goal is to specify a reliable mechanism based on which the sensor nodes will decide on which storage node they will send the data of the object they detected within their coverage area, aiming to maximize the location privacy ensured. Therefore, the primary objective is the creation of a data dissemination protocol between sensor and storage nodes to increase the level of location privacy. In this respect, it is essential to partition the sensor nodes into groups based on certain criteria and constraints. A significant matter that supports such a decision is the determination of the privacy-endangering distance range between the sensor nodes that is caused by potentially partially overlapping sensing areas. Therefore, if *r_d_* is the detection range of the sensor nodes of the network we define as privacy-endangering distance range the distance range [2*r_d_* − *α*, 2*r_d_*] between the sensor nodes, where α > 0 is a configurable privacy parameter that can be adapted based on the privacy preservation requirements of the specific system and is determined before the beginning of the monitoring phase. The concept behind this is that the smaller the intersection of the coverage areas of two sensor nodes, the more accurately a potential intruder can identify the location of the targeted object if the object detection messages of both these nodes are intercepted. For sensor node distance higher than 2*r_d_* this intersection is null and there are no privacy concerns. But if their distance is equal to 2*r_d_* − *ε*, the larger the positive quantity *ε* is, the larger the respective coverage area intersection is. The difference between the privacy parameter α and variable ε is that α constitutes a configurable parameter important for the privacy level of the network while ε refers to the relation between the distance of sensor nodes and the overlapping area The detection nodes positioned within this distance range should thus not send their detection data to the same storage node, as an intruder could exploit the limited common sensing area to infer sensitive information concerning the location of the mobile target. Therefore, the set *Nbr* of neighboring sensor node pairs is defined, which is actually the set of any pair of sensor nodes (*x_i_*, *x_i_*_’_) that are positioned within range [2*r_d_* − *α*, 2*r_d_*]. The wider the area of the *I*-state of a storage node is, the more difficult it is for an intruder to deduce accurate location information. In this framework, the research work presented herewith aims to specify a mechanism that will result in properly clustering all sensor nodes *x_i_*, where *I* = 1, 2, …, *n*, and allocating each of them to a specific storage node *y_j_*, where *j* = 1, 2, …, *m*. The resulting function can be specified as follows: *C*(*x_i_*, *t*) = *y_j_*, which suggests that under the conditions of the sensor network of the IoT environment at time t, sensor node *x*_i_ is allocated at storage node *y_j_* and will thus post only to this specific storage node any object detection message generated.

### 4.2. Evaluation Metrics

Three evaluation metrics are defined to measure the effectiveness of the protocols that will be proposed aiming to protect the privacy of location information across the IoT infrastructure. These metrics are defined as follows:

*Privacy*: This metric is based on the respective performance criterion introduced in [2]. Assuming an attacker has violated a storage node *y_j_* and managed to access any information available at this node, the value of privacy *P_r_*(*t*) at any given moment *t* is expressed by the percentage of data that is still safe and has not been compromised. The attacker has access to location data corresponding to $LA_{j}\left(t\right)$, the potential location area of storage node *j* that is compromised, while the location information of the entire IoT infrastructure is expressed by the location area $LA^{*}\left(t\right)$ identified by the global node. Assuming the worst case scenario, where the intruder has obtained access to the data of the storage node corresponding to the smallest possible potential location area, the value of privacy $P_{r}\left(t\right)$ at a given moment *t* is expressed as follows:

(1) $$P_{r}\left(t\right)=1-\frac{LA^{*}\left(t\right)}{\underset{j}{\min}\left\{LA_{j}\left(t\right)\right\}},\;j=1,2,\ldots ,m$$

As one may easily observe, as $LA_{j}\left(t\right)$ decreases and approaches the value of $LA^{*}\left(t\right)$, the value of $P_{r}\left(t\right)$ decreases as well, while the case where $P_{r}\left(t\right)=0$ corresponds to the situation where a single storage node has access to the knowledge of the entire IoT infrastructure. On the other hand, $P_{r}\left(t\right)=1$ corresponds to full privacy, but in the problem studied herewith a security breach to a single storage node is assumed and thus, this value can never be achieved.

The research presented in this paper is based on the assumption that an intruder can compromise up to 1 storage node. However, the proposed approach can be generalized to addressing the case of multiple storage nodes being compromised [33]. To enable this, the metric of privacy should be modified to reflect the possibility of data breach from up to *q* storage nodes. The set j=1,2,…,m should be replaced by set $j^{\prime }=\left\{\left(1\right),\left(2\right),\left(3\right),\ldots \left(1,2\right),\left(1,3\right),\ldots ,\left(1,2,3\right),\ldots ,\left(1,2,3,\ldots ,q\right)\right\}$, where *q* < *m*. Thus, the set j’ constitutes the power-set of set j with up to *q* storage nodes being comprimised. To achieve this, the denominator of the privacy $P_{r}\left(t\right)$ fraction needs to be adjusted, as $\underset{j}{\min}\left\{LA_{j}\left(t\right)\right\}$ refers to the minimum area of a state that a single storage node demonstrates. Thus, $\underset{j}{\min}\left\{LA_{j}\left(t\right)\right\}$ should be replaced by min{*LA*_1_(*t*),*LA*_2_(*t*),*LA*_2_(*t*),…,*LA*_1_(*t*)∩*LA*_2_(*t*),*LA*_1_(*t*)∩*LA*_3_(*t*),…,*LA*_1_(*t*)∩*LA*_2_(*t*)∩*LA*_3_(*t*),…,*LA*_1_(*t*)∩*LA*_2_(*t*)∩*LA*_3_(*t*)∩…∩*LA_q_*(*t*)}.

*Conflict*: As conflict in this research, we define a Boolean variable that indicated whether or not two sensor nodes that are positioned within dangerous distance range [2*r_d_* − *α*, 2*r_d_*], are allocated to the same cluster and thus send their data to the same storage node. The conflict metric at a given time *t* that is estimated for any pair of sensor nodes $x_{i}$ and $x_{i^{\prime }}$ in the IoT infrastructure can be expressed as follows:

(2) $$Conflict\left(x_{i},x_{i^{\prime }},t\right)=\{\begin{array}{cc} 1, & C\left(x_{i},t\right)=C\left(x_{i^{\prime }},t\right)\;\mathrm{and}\;\left(x_{i},x_{i^{\prime }}\right)\in Nbr\\ 0, & \mathrm{otherwise} \end{array},$$

where $C\left(x_{i},t\right)$ is the cluster where sensor node $x_{i}$ is assigned to at time *t*. Apparently, if the pair of $\left(x_{i},x_{i^{\prime }}\right)$ of sensor nodes is included in the set *Nbr* of neighboring sensor node pairs defined in Section 4.1, then once these two nodes are assigned to the same cluster at a given time *t*, the following stands: $Conflict\left(x_{i},x_{i^{\prime }},t\right)=1$. On the other hand, for any sensor node pair that is not included in set *Nbr*, the value of this metric is always $Conflict\left(x_{i},x_{i^{\prime }},t\right)=0$. As one may easily observe, the smaller the value of the sum of all these conflict indicators is, the higher the protection of the location privacy across the IoT infrastructure is.

*Average Privacy*: This metric is denoted by $\bar{P_{r}\left(T\right)}$ and it reflects the level of location privacy achieved throughout a given time period *T*, where the mobile target object moves within the range of the IoT infrastructure. It can be expressed as follows:

(3) $$\bar{P_{r}\left(T\right)}=\frac{1}{T}\int_{T}P_{r}\left(t\right)dt.$$

Apparently, since $P_{r}\left(t\right)\in \left[0,1\right)$, ∀t, the same also stands for $\bar{P_{r}\left(T\right)}$, i.e., $\bar{P_{r}\left(T\right)}\in \left[0,1\right)$, ∀T. Since a single value for the average privacy metric is calculated for each entire experiment, this metric is a quite reliable evaluation criterion that can be used to compare the performance of the various algorithms for a given experiment.

### 4.3. Formal Problem Statement

Based on the problem concepts and setting presented, as well as the definition of the evaluation metrics introduced in the previous subsections, the studied problem can formally be stated as follows.

Assume the case of a mobile object the location of which is monitored by an IoT infrastructure. This infrastructure has *n* fixed sensor nodes denoted by $x_{1},x_{2},\ldots ,x_{n}$, that are of equal object detection range $r_{d}$. Assume a malicious intruder that can intercept the location data of up to one storage nodes. 

Given the above, (1) specify a sensor node clustering function $C\left(x_{i},t\right)=C_{k}$, k=1,2,…,K that assigns each node $x_{i}$ to a specific cluster $C_{k}$, so that no conflicts exist for any pair of the given sensor nodes $x_{1},x_{2},\ldots ,x_{n}$, i.e.,:

(4) $$C\left(x_{i},t\right)\neq C\left(x_{i^{\prime }}t\right)\;\forall \;\left(x_{i},x_{i^{\prime }}\right)\in Nbr,$$

and so that the number *K* of the distinguished clusters is minimum, in the sense that there is no clustering approach that can eliminate all conflicts distinguishing *K* − 1 clusters across the given sensor nodes, and (2) also given the population *m* of the storage nodes in the IoT infrastructure denoted by $y_{1},y_{2},\ldots ,y_{m}$, specify a second sensor node clustering function $C^{\prime }\left(x_{i},t\right)=y_{j}$, j=1,2,…,m that assigns each node $x_{i}$ to a specific storage node $y_{j}$, so that the overall number of conflicts $\sum_{i}\sum_{i^{\prime }}Conflict\left(x_{i},x_{i^{\prime }},t\right)$ ($\forall i,i^{\prime }\in \left\{1,2,\ldots ,n\right\}$) that exist for all pairs of the given sensor nodes $x_{1},x_{2},\ldots ,x_{n}$ is minimum, i.e.,:

(5) $$\underset{C^{\prime }\left(x_{i},t\right)}{\min}\left\{\sum_{i}\sum_{i^{\prime }}Conflict\left(x_{i},x_{i^{\prime }},t\right)\right\},\;\forall i,i^{\prime }\in \left\{1,2,\ldots ,n\right\},$$

aiming to minimize the amount of location information of a specific object stored to the same storage node across the IoT sensor network.

Since a single value for the average privacy metric is calculated for each entire experiment, this metric is a quite reliable evaluation criterion that can be used to compare the performance of the various algorithms for a given experiment.

## 5. Proposed Solution

### 5.1. The Dynamic Clustering Algorithm (DCA)

The basic idea of Dynamic Clustering Algorithm (DCA) is the fact that the sensor nodes, which have been classified in the same cluster as the ones that are positioned within dangerous distance from one another, can dynamically change the cluster they belong to and send their data to a different storage node depending on the location of the mobile target. This technique is based on fractional coloring methods [34,35] which consequently improve the IoT sensor network security level with respect to the traditional graph coloring [36,37]. In the remainder of this section the DCA is introduced and described in detail.

#### 5.1.1. DCA Phase 1: Initial Clustering

The first step of the method is the static network analysis and the initial cluster identification. Three clustering algorithms are used (i.e., the constrained agglomerative hierarchical algorithm, the PCk-means and the walkthrough algorithm [38]) to group the sensor nodes in *k* clusters. Number *k* indicates the minimum population of clusters that ensures there are no conflicts between the sensor nodes of the network. Number *k* is actually determined by the constrained clustering algorithms used and is not one of the input parameters for these algorithms. However, since it is estimated prior the execution of the DCA, it does not affect its performance. The main goal of the node clustering is to ensure that all nodes *x*_i_ will be assigned to different clusters than their neighbors *x_j_*, in case their distance lies within interval range [2*r_d_* − *α*, 2*r_d_*], where α > 0 is a configurable privacy parameter and *r_d_* is the detection range of the sensor nodes of the network, as defined in Section 4.1. 

**Figure 2:**
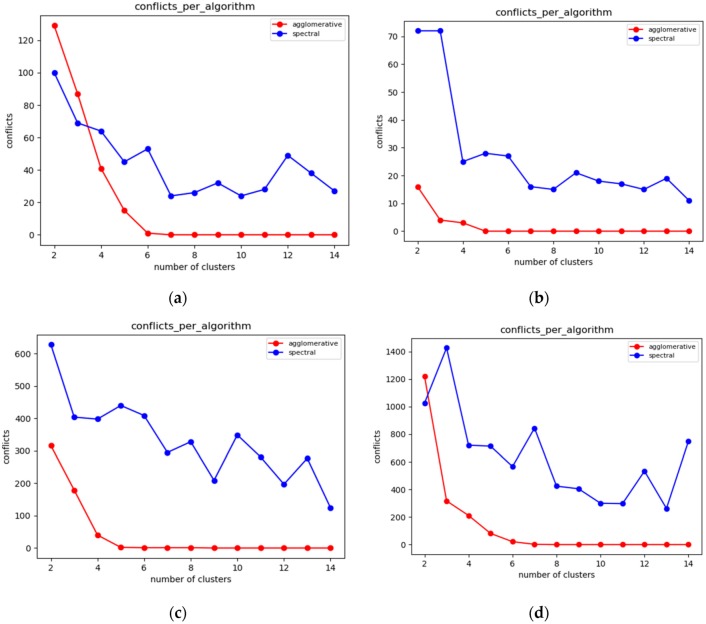
Comparison between constrained hierarchical agglomerative algorithm and constrained spectral algorithm (**a**) 37 nodes, *r_s_* = 3, *a* = 2, *ann* = 9.2, (**b**) 100 nodes, *r_s_* = 3, *a* = 0, *ann* = 1.4, (**c**) 100 nodes, *r_s_* = 4.5, *a* = 2.6, *ann* = 22.6, (**d**) 123 nodes, *r_s_* = 9, a = 8.2, *ann* = 29.9.

Additionally, the minimum number of conflicts for a given number of storage nodes is estimated in this DCA phase and the results that can be achieved by the selected algorithms are aggregated. The algorithms implemented and empirically matched to this type of networks are the walkthrough [2], constrained hierarchical agglomerative algorithm [39,40], pck-means [39,40], which are described in Appendix A. These algorithms have been selected among other popular algorithms, such as constrained spectral clustering [39,40], cop kmeans [39,40], etc., once initial experimentation indicated that they are more stable regarding the overall number of conflicts imposed. As depicted in Figure 2, constrained spectral clustering is not even comparable with constrained hierarchical agglomerative algorithm and that is why it was excluded from the selection of the algorithms. Cop kmeans constitutes a greedy algorithm that does not allow even one of the constraints to be violated. For this reason, it does not match with our concrete problem. The number of clusters is selected according to the IoT network characteristics, the number of sensor nodes and the respective sensor network density. Once the initial sensor node clustering is performed, each cluster is bound to a specific storage node. 

In most cases however, the number *m* of storage nodes, is (much) smaller than the minimum number of clusters *K*, for which there are zero conflicts, so either more than one clusters need to be assigned to the same storage node, or there are clusters are not assigned to any storage node. Therefore, in the case where *m* < *K*, then dynamic clustering of the sensor nodes is required to minimize the overall number of conflicts. This step initiates by assigning each storage node to a specific cluster. All sensor nodes are aware of the id of the nodes that are located at distance within the range of [2*r_d_* − *α*, 2*r_d_*] from them, as well as of the cluster they have been assigned to. There are three ways based on which the sensor nodes that will be reassigned to another cluster are dynamically selected:

*Selective*: The sensor nodes are grouped into *m* cluster, where *m* is equal to the number of storage nodes. By examining one by one the pair of nodes, the distance of which lies within the range [2*r_d_* − *α*, 2*r_d_*], the pairs of such neighboring nodes that belong to the same cluster are identified. Cluster number *m* + 1 is assigned to one of the two nodes of each of these pairs.

*Direct*: The *K* clusters are sorted by sensor node population and the *K* − *m* smaller ones are selected. The nodes of these *K* − *m* clusters are assigned to cluster *m* + 1, while the rest of the *m* clusters are assigned to one storage node each.

*Hybrid*: This approach of selecting the sensor nodes to be reassigned to another cluster is a combination of the previous two. The clusters are sorted by sensor node population. There are *K* − *m* + 1 ways to divide the nodes into valid clusters, where *K* is the minimum cluster population where the sum of conflicts is equal to zero and *m* is the number of storage nodes. If *K* = *m*, this method is identical to Selective. If *K* > *m*, the Direct method is used and the sensor nodes of the *K − m* smallest clusters are merged to a single cluster identified as cluster *m* + 1. 

#### 5.1.2. DCA Phase 2: Preprocessing Stage

Irrespective of the method selected in Phase 1, we end up with *m* + 1 clusters, where only the last cluster includes neighboring nodes that introduce conflicts. The sensor nodes of this cluster are considered to be unclassified, initially not being assigned to any of the storage nodes. Ideally, each of these will be dynamically allocated to another cluster, aiming to minimize the conflicts among the sensing nodes able to detect the moving object at the same time *t*. The second phase of the DCA corresponds to an intermediate preprocessing step, during which the sensor nodes of *m* + 1 cluster that can be moved to one of the first *m* clusters are identified, without any conflicts being created, given the location of the mobile object at the given time. As far as the communication cost of the algorithm in this phase is concerned, each sensor node that moves from cluster *m* + 1 to another existing cluster, broadcasts its new cluster number to its neighbors, some of which are assigned to cluster *m* + 1. Thus, the overall number of the messages being exchanged depends on the population of sensors assigned to *m* + 1 cluster and the average number of neighbors that they have.

#### 5.1.3. DCA Phase 3: Dynamic Allocation

The final phase of the DCA is the core of the dynamic clustering, so if at a specific time *t*, the number of neighboring sensors that are detecting the moving target is *d*, where *d* < *m*, then node *x_i_* that is assigned to cluster *m* + 1 can be assigned to one of the *m-d* remaining clusters. If *d* > *m*, node *x_i_* selects the cluster of the least used storage node by counting the number of currently positively detecting sensor nodes that send their sensed location data to this storage node. DCA introduces the concepts of “message of interest” and “message of no interest” in order to be more energy efficient. When a sensor node detects the target object for the first time and does not belong to cluster *m* + 1, it sends a message of interest to each sensor node that belongs to the newly created cluster that also lies among its neighbors. Thus, the messages of interest inform the nodes of dynamic clustering that some nodes are going to participate in the detection process. When this specific node does not detect the mobile target anymore, it sends a message of no interest to its neighbors. In this way, the population of the messages being exchanged is reduced as notifications are posted only when a sensor starts or stops detecting the mobile node and not during the entire location detection process. Therefore, the respective population of exchanged messages depends not only on the number of average neighbouring nodes in the IoT infrastructure, but also on the velocity of the mobile object and its exact trajectory. The precise location of the mobile node is always accessible via the Global node that aggregates the *LA_j_*(*t*) state of storage nodes at any time *t*. The algorithm applies only to the distribution of data among sensor and storage nodes and does not affect the information that maintains the Global node. Below are the steps of the algorithm accompanied presented in the form of pseudocode.


**Dynamic Clustering Algorithm (DCA).**
Input:  *K*: minimum number of clusters for which there are no conflicts  *i*: indicates the sensor node that executes the algorithm  size[*k*1, *k*2, …, *kK*]: the population of nodes that each cluster includes  Cluster[C1, C2, …, C*n*]: for each of the *n* sensor nodes it indicates the number of clusters the respective sensor belongs to  *m*: indicates the number of expected cluster population  DDist[(a, b), (a, c)…, (d, w)]: Pairs of nodes positioned in dangerous distance range Output: Clustering that is adapted according to the target position  1: sorting (size) //sorting clusters by size  #1st phase:  2: If strategy = ”direct”:  3: If Cluster[*i*] <= *m*: Continue  4: Else: Cluster[*i*] = *m* + 1  5: Else if strategy = ”selective”:  6: For each link ∈ DDist:  7:  *t*1 = link[0]  8:  t2 = link[1]  9:  If Cluster[*t*1] = Cluster[*t*2]: Cluster[*t*1] = *m* + 1  10: Else:  11: Steps 6–9 //hybrid  12: Steps 3–4  #2nd phase:  13: If cluster[*i*] = *m* + 1:  14: *d* = 0  15: For each link ∈ Nbr: //each sensor knows its neighbors  16: If Cluster[link] is not used from neighbors:  17: *d* = *d* + 1  18: If *d* < *m*:  19: Choose an unused cluster number  #3rd phase:  #Input:  #target: position of mobile target at time *t*  #counters[*q1*, *q2*, …, *qn*]: counting how many sensor nodes send data to specific storage node  20: If |target-*xi*| <= *rd*:  21: If Cluster[*i*] < *m*+1:  22: Send message to corresponding server  23: For each *xi* ∈ *Nd*:{C(*xi*) = *m*+1}:  24:  If it is the beginning of detection:  25:  Send message of interest to *xi*  26:  If it is the end of detection:  27:  Send message of not interest to *xi*  28: Else:  29: For each *xi* ∈ *Nd*:  30: *d* = 0  31: If cluster number is unused:  32:  *d* = *d* + 1  33: If *d* < *n*:  34: Choose one of the rest  35: Else:  36:   index = min(counters) // server which is least used  37: Send message to corresponding server

It is important to highlight at this point that DCA phases 2 and 3 are required for distributed execution of the algorithm of the algorithm, which means that all nodes execute these phases simultaneously. Each sensor node has its own sets *Nbr* and *Nd* and is aware of them before the execution of the algorithm. As already stated, *Nbr* consists of all pairs of sensor nodes that are located within distance that lies in the range of [2*r_d_* − *α*, 2*r_d_*], while *Nd* carries all pairs of sensor nodes that are up to 2*r_d_* away. The position of the mobile target is assumed to be constantly changing, as respective object is moving across the IoT sensor network. Each counter corresponds to an array that each sensor node carries, capturing (per storage node) the population of its neighbors that send the location data they monitor to the specific storage node, while *xi* refers to the position of the corresponding sensor node.

The algorithm introduced above differs from the static clustering of conventional algorithms such as constrained hierarchical clustering and PCk-means in a way that adapts its behavior based on the trajectory of the target. It is more flexible than traditional clustering algorithms as it constantly redirects the data between sensor and storage nodes to maximize privacy.

### 5.2. Advantages of the DCA

This subsection aims to illustrate the improvements that DCA can yield by elaborating on a specific use-case over an existing deployed topology. The use-case assumes that the cluster numbers correspond to colors and that there are three storage nodes in the IoT infrastructure, i.e., there are three available colors to be assigned to the sensor nodes in place. It is assumed that there are 4 sensor nodes that are positioned on the angles of a perfect square of known side length. Thus, there is no free color for the fourth node, which is forced to choose one of the colors that are already used. If the red color is used by the fourth node, privacy endangering area *a*(34,234,134,1234) (this area *a* for example represents the union of region 34 (i.e., the region where the mobile object is detected simultaneously only by sensor nodes 3 and 4), region 234, region 134 and region 1234.) is observed, if the yellow color is selected, privacy endangering area *β*(14,134,124,1234) is observed, while in case the brown color is selected, privacy endangering area *γ*(124,234,1234) is observed, thus imposing a privacy problem in all alternatives. Subsequently, it is illustrated how the DCA outperforms the classical graph coloring theory.

**Figure 3:**
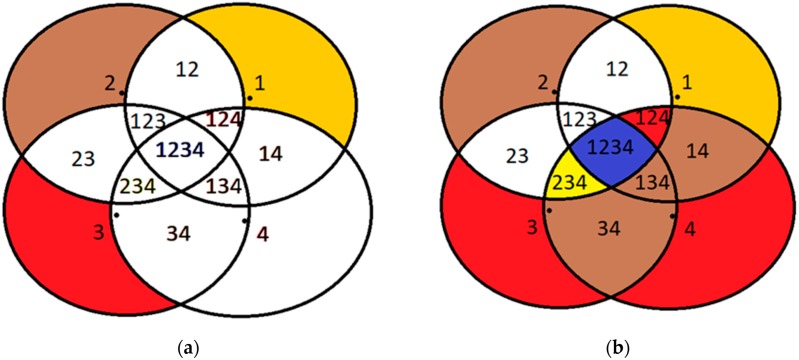
(**a**) Conventional Graph coloring, (**b**) implementation of DCA.

The first phase of the DCA is used to find the minimum number of colors *K* that results in zero overall conflicts in the sensor network. In this use-case, the DCA identifies *K* = 4 and assigns the colors of yellow, brown, red (*m* = 3) to sensor nodes 1, 2, 3 while node 4 is assigned to the non-existing color *m*+1 = 4 that is white, as depicted in Figure 3a. Thus, the classical graph coloring theory has properly been employed since no sensor node has the same color as a neighboring one. However, while the first 3 nodes are assigned to an existing storage node, the fourth node cannot send its messages to an existing storage node.

Based on the implementation of DCA, node 4 dynamically changes its color considering the position of the moving target and on the neighboring sensor nodes that detect this at the same time. Thus, it selects one of the available colors (and therefore one of the storage nodes), which are not used at that specific time. For example, in area 4 because no sensor node detects the mobile object other than node 4, the location information messages of node 4 can be directed to any storage node. In region 134 the only node that does not detect the mobile object is node 2, in region 14 nodes 2 and 3, and in region 34 nodes 1 and 2. In this case, where the mobile object is located in any of these three regions, color 2 (brown) is assigned to node 4 by the DCA that is not used by any of the sensor nodes detecting the mobile object at that time. Similarly, in region 124, color 3 (red) is assigned to node 4 (since node 3 does not detect the object), while in area 234, node 4 is assigned to color 1 (yellow) for the same reason. Of particular interest is the blue area of the figure where all nodes simultaneously detect the moving target and all colors are used. In this area, it is not possible to assign any color to node 4 that will eliminate the conflicts, and thus, node 4 arbitrarily selects any of the *m* colors (and a storage node respectively) to send the location data it senses. A slight improvement that is made here is that node 4, being aware of the exact positions of its neighboring sensor nodes, selects the color of its closest neighbor because the intersection of their respective coverage areas is the largest possible, thus disclosing location information of lower granularity. This is valid as for example if sensor node 4 is assigned to color 3 (red), a potential intruder that compromises storage node 3 can deduce that the mobile object is located in area 34,134,234,1234, which is surely larger than area 124,234,1234, that would be deduced by an intruder compromising storage node 2, in case sensor node 4 is assigned to color 2 (brown).

In a nutshell, as one may easily observe, even though there is a single area in the IoT sensor network (region 1234), where the conflicts cannot be eliminated for any allocation of the three colors to the four sensor nodes, in all other areas the DCA achieves in eliminating the overall conflicts, thus outperforming the static color assignment, as presented in Figure 3b. In the first case, the problematic areas include *a*(34,234,134,1234), or *b*(14,134,124,1234), or *c*(124,234,1234), which are all much larger than the only problematic area resulting by the DCA (i.e., region 1234).

If we had a static clustering based only on the distances, the decision of the node regarding to which storage node would send its data, would be affected by sensors that do not participate in the monitoring procedure at this specific moment. However, with this technique, the sensor node that detects the mobile target takes into consideration only those which detect the same moment t. Thus, a sensor node can adapt its decision based on the trajectory that the mobile node follows instead of sending its data always to the same storage node. In most cases, a storage node will be used less than the others for a specific period allowing some sensor nodes to send the messages to it. In this way, the area where there are no available storage nodes is narrowed down dramatically. However, if the mobile object enters the problematic area, the improvement of DCA, which is the utilization of the least used storage node, leads to the increase of privacy level of the network.

### 5.3. Generalisation to Address Multiple Mobile Objects

The DCA proposed in this paper can be extended to integrate multiple objects. When more than one mobile target is monitored by the sensor network, independent and identical dynamic clustering approaches can be employed for each one of them. In this case different instances of the studied problem are addressed independently. The first phase of the DCA is the same for all mobile objects as the initial clustering is executed to compute the number of conflicts of the network itself based on the distances between the sensor nodes of the IoT infrastructure. The second and third phase of the DCA that constitute the distributed parts of the algorithm, are executed for each mobile object independently. Both storage nodes and Global node maintain distinguished values of $LA_{jl}\left(t\right)$ and $LA_{l}^{*}\left(t\right)$ states for each mobile object *l*. Moreover, the sensor nodes are able to distinguish the messages that they send for every mobile object. The metrics of Privacy and Average Privacy are computed separately and independently for each mobile target. However, it is expected that the power consumption of the sensor nodes will increase as these will now be required to send a higher number of messages, while the storage nodes should engage more memory resources to maintain the different $LA_{jl}\left(t\right)$ states.

## 6. Experiments and Evaluation

### 6.1. Experiments’ Setting

**Figure 4:**
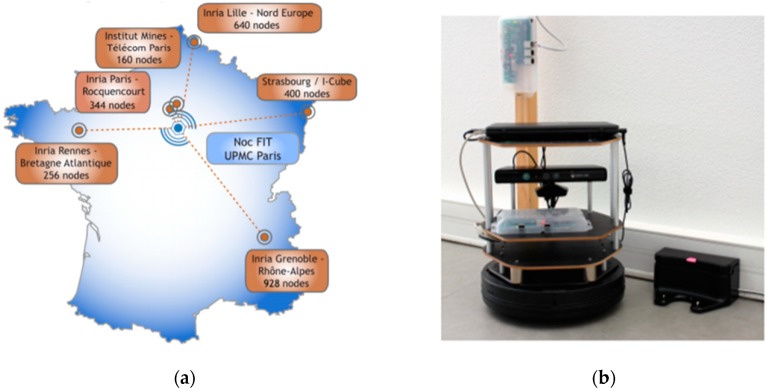
(**a**) FIT IoT LAB infrastructure, (**b**) Turtlebot.

The Future Internet Testing—Internet of Things Laboratory (hereafter referred to as FIT IoT LAB) is an infrastructure that is made available for researchers that wish to experiment over an IoT infrastructure with wireless sensor nodes and various heterogeneous devices [41,42,43]. FIT IoT LAB includes over 2000 presence detection devices that are deployed in six different locations in France. More specifically, it consists of 2728 wireless sensors nodes deployed in Inria Grenoble (928), Inria Lille (640), ICube Strasbourg (400), Inria Rocquencourt (344), Inria Rennes (256) and Institut Mines-Telecom Paris (160) as shown in Figure 4a. It is essentially a scientific testbed that provides full control over wireless devices and access to libraries that help researchers to extract various information about the sensor nodes deployed, such as power consumption, end-to-end delays, and congestion. It facilitates the network deployment as well as the process of collecting and analyzing data.

But beyond the static detection nodes, FIT IoT LAB provides a number of mobile nodes. So far, it has 15 mobile hubs in production in the following locations: Grenoble (2), Lille (3), Strasbourg (10). These nodes have predetermined trajectories and move on the ground across the static sensor nodes. The mobile target of the experiment was a turtlebot node provided by FIT IoT LAB. It consists of a notebook, a Kinect, a FIT–IoT node and a gateway as depicted in Figure 4b. The notebook is used to support communication with the FIT IoT LAB infrastructure via wifi and to control the robot through the ROS operating system [44,45,46]. The mobile node is accessible in exactly the same way as the static nodes of the IoT infrastructure through API.

As already mentioned, the mobile node trajectories are predetermined and are different for each location (Strasbourg, Lille, Grenoble). The trajectory of the mobile node, when completed, is repeated several times until the required time of the experiment is reached. It also has the ability to bypass the obstacles. As far as the technical characteristics of the turtlebot are concerned, it has a maximum speed of 0.7 m/s, which is represented by *v*_max_ and is used to configure the DCA in the experiment execution. It is equipped with two batteries, of up to three h lifetime when fully charged and can be recharged in six h. The coordinates of the mobile track are recorded.

### 6.2. Experiments’ Execution

**Figure 5:**
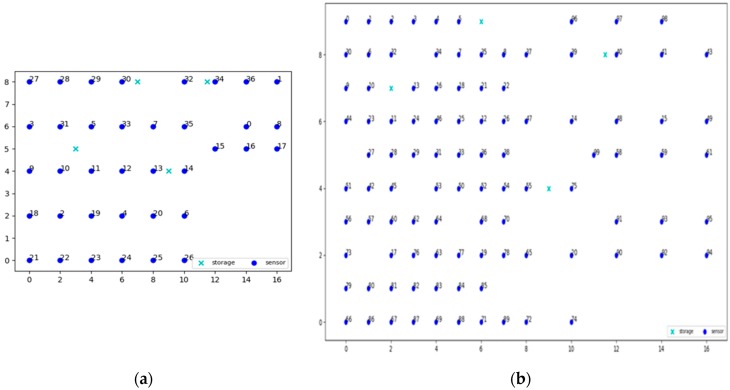

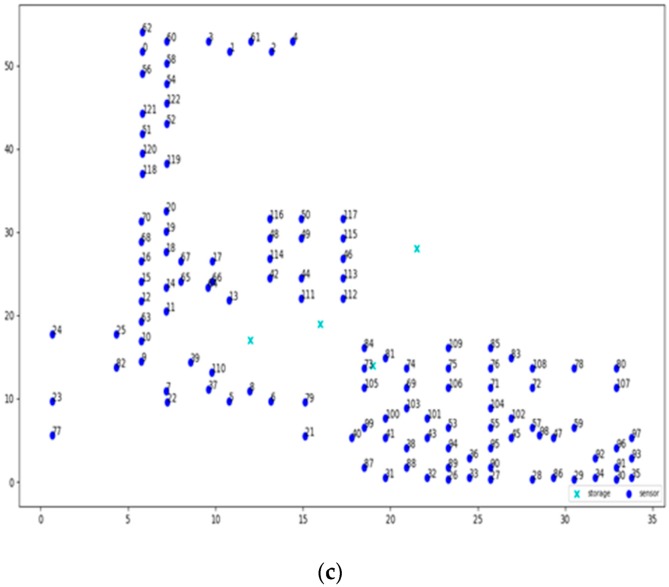
The topologies of experiments as provided by FIT-IoT lab (**a**) 37 nodes, (**b**) 100 nodes, (**c**) 123 nodes.

**Figure 6:**
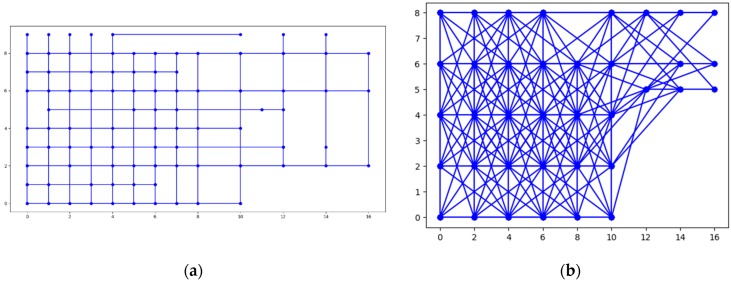

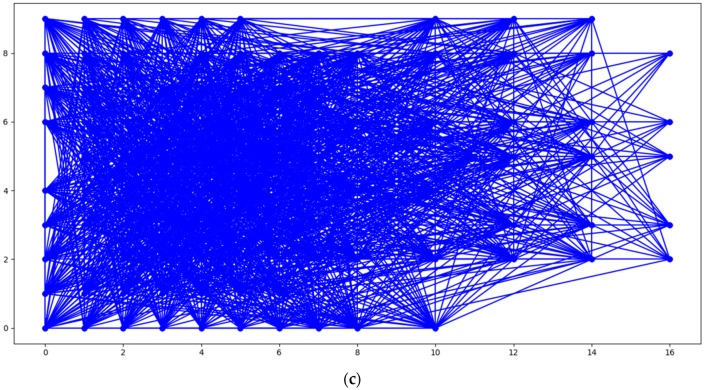
Represents the density graph where the sensor nodes are the vertices and the edges connect those whose distance belong in dangerous distance area (**a**) sparse graph with *ann* = 1.4, (**b**) medium dense with *ann* = 9.2 (**c**) very dense with *ann* = 22.6.

Three different topologies from the FIT IoT LAB have been used to for the needs of the evaluation experiments conducted. These three topologies are depicted in Figure 5 and engage 37, 100, or 123 sensor nodes, respectively. To distinguish sensors from storage nodes they are displayed in different color and using a different symbol. The sensor nodes are displayed as dark blue circles, while the storage nodes are displayed as light blue crosses. These three topologies have been used to conduct eight static clustering experiments to evaluate and compare the performance of the pck-means, walkthrough and constrained hierarchical agglomerative algorithms for different populations of storage nodes *m*. Different values have been used for the detection range of sensors *r_d_*, as well as for the privacy parameter *a* to change the nodes that detect the mobile target for any possible position over the area covered, as presented in Section 6.3. As a criterion for the density of the topology the average population of neighbors is used, i.e., the population of sensor nodes that are positioned in dangerous distance range. The higher the average number of neighbors (*ann*) for each sensor node is, the more dense the topology is, as illustrated in Figure 6. For the experimental evaluation of the dynamic clustering, the same topologies have been used, but also introducing moving targets, i.e., turtlebots of 0.7 m/s maximum velocity, which followed the predetermined trajectories supported by the FIT IoT LAB that are studied in Section 6.4. The performance of the DCA in dynamic settings has been compared to the performance of the walkthrough algorithm via the privacy metric that is evaluated in Section 6.4. Using the FIT IoT LAB facilities, we took a snapshot of the moving target track per 0.1 s and recorded the corresponding *x*, *y* location coordinates. In this framework, 12 experiments have been conducted where the sensor nodes have been dynamically clustered based on the current location of the mobile object. In each experiment, the number of the storage nodes varied, along with the detection radius *r_s_* and/or the privacy parameter α. These various configurations enabled us to explore how the DCA behaves as the population of the network storage nodes increases and its density increases or decreases. Thus, the performance of the proposed algorithm has been evaluated in different topologies, under various conditions. The evaluation results obtained for both dynamic and static clustering of the sensor nodes are presented hereafter by topology.

### 6.3. Static Clustering Evaluation

In this section, the evaluation results obtained by the experiments regarding the static clustering delivered based on the walkthrough, the constrained hierarchical agglomerative clustering and the PCk-means algorithms before introducing the mobile target. The diagrams that follow depict the overall number of conflicts that each algorithm achieves over varying populations of storage nodes (and clusters respectively).

#### 6.3.1. Experiments over 37 Node Topology

**Figure 7:**
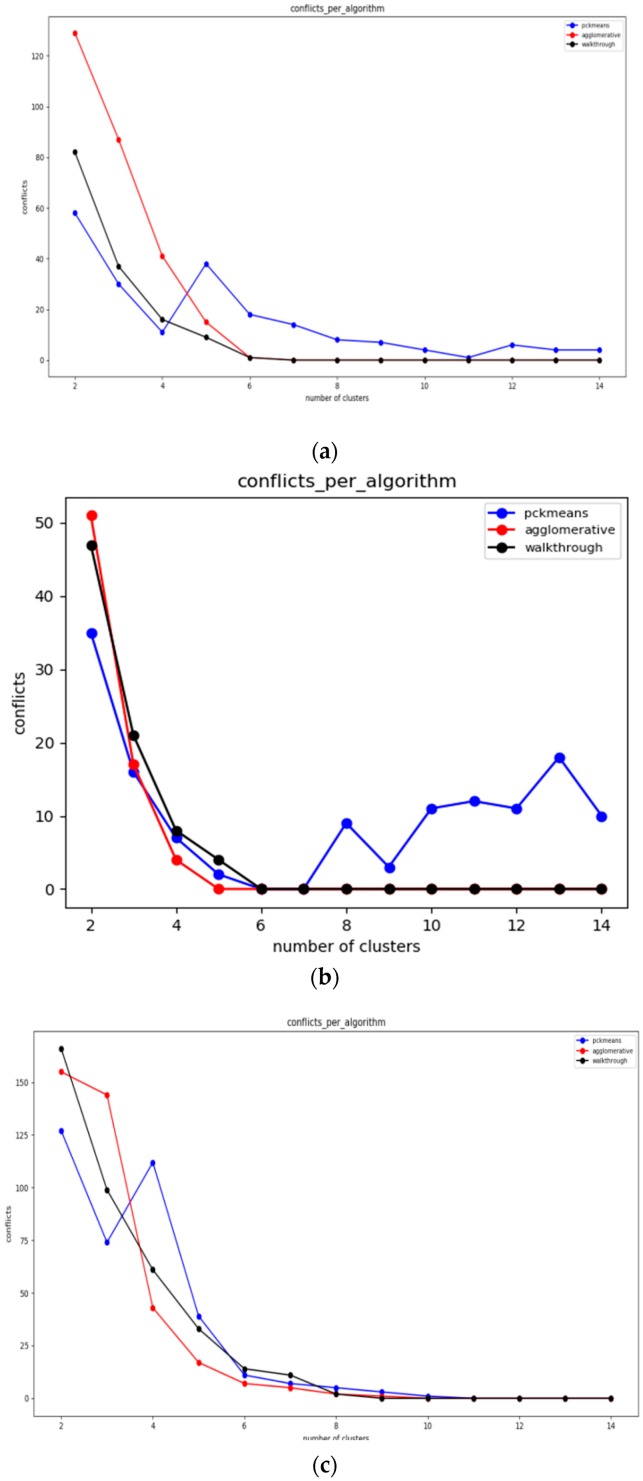
Evaluation results for static clustering over the topology of 37 nodes (**a**) *r_s_* = 3, *a* = 2 and *ann* = 9.2, (**b**) *r_s_* = 1.5, *a* = 2 and *ann* = 5.7 (**c**) *r_s_* = 4.5, *a* = 4.7, and *ann* = 17.8.

Given the above experiments, we can clearly deduce that pck-means achieves the lower number of conflicts in all cases for *K* < 4. The first stage of pck-means takes into consideration the transitive closure of the constraints and initializes the centroids appropriately. However, it is an unstable algorithm as it cannot consistently achieve lower number of conflicts as the overall population of clusters *K* increases. One can also easily observe in Figure 7a that refers to a medium dense topology that the walkthrough algorithm performs far better than the constrained agglomerative hierarchical algorithm. Figure 7b corresponds to a sparse topology where the algorithms achieve similar results, while Figure 7c represents a very dense one where the walkthrough algorithm is outperformed in all cases except for *K* = 2.

#### 6.3.2. Experiments over 100 Node Topology

**Figure 8:**
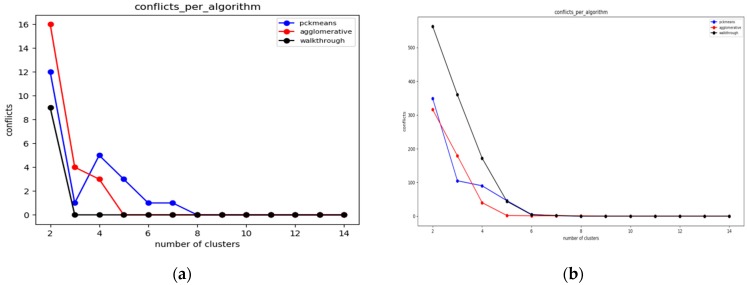
Evaluation results for static clustering over the topology of 100 nodes for (**a**) *r_s_* = 3, *a* = 0 and *ann* = 1.4, (**b**) *r_s_* = 4.5, *a* = 2.6 and *ann* = 22.6.

The settings of the experiments herewith aim to enable the comparison between a sparse sensor network topology (Figure 8a) and a quite dense one (Figure 8b). In Figure 8a, one may easily observe that the walkthrough algorithm performs much better than the other two in sparse topologies, while in Figure 8b the constrained hierarchical agglomerative algorithm appears to be more suitable for dense topologies.

This is reasonable as hierarchical algorithms aggregate any two clusters based on some predetermined criteria, while walkthrough is a greedy and distributed algorithm that attaches cluster numbers to each node based on the clusters available. Thus, the more the constraints that need to be met are, the more difficult for the walkthrough is to find available clusters. The performance of pck-means is less stable than those of the other two and particularly for the sparse topology it is greatly outperformed by the walkthrough algorithm for low cluster populations.

#### 6.3.3. Experiments over 123 Node Topology

**Figure 9:**
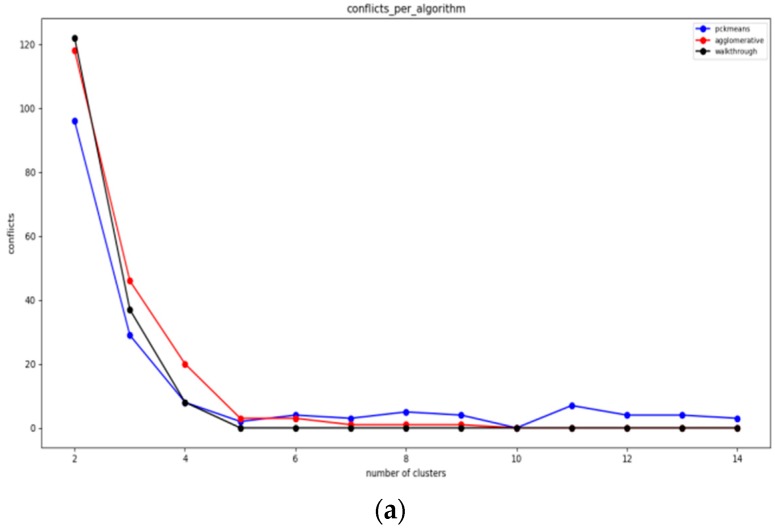

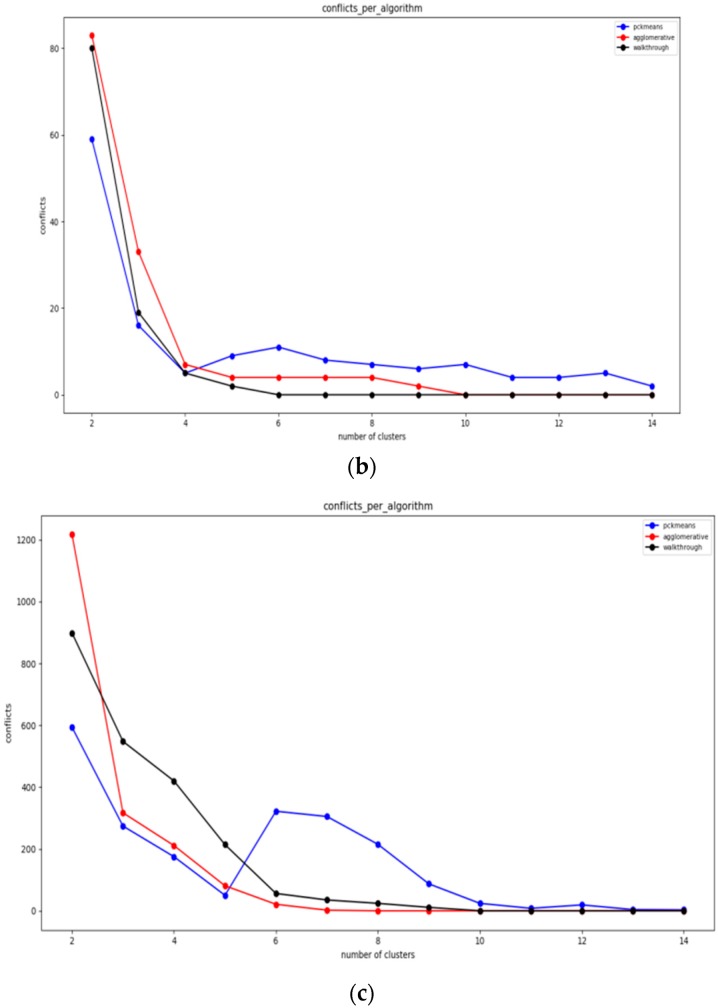
Evaluation results for static clustering over the topology of 123 nodes for (**a**) *r_s_* = 3, *a* = 2 and *ann* = 5.2, (**b**) *r_s_* = 1.5, *a* = 2 and *ann* = 3.5, (**c**) *r_s_* = 9, *a* = 8.2 and *ann* = 29.9.

In Figure 9a,b, which correspond to quite sparse sensor network topologies, the walkthrough algorithm outperforms the other two for *K* > 4. In Figure 9c, where the experiments have been conducted over a very dense network topology, the constrained hierarchical agglomerative algorithm is more suitable for *K* > 5. On the other hand, one may easily observe the advantages of the pck-means regarding the initialization of centroids as it achieves the minimum number of conflicts for *K* < 6.

#### 6.3.4. Overall Evaluation Results

Each algorithm has advantages and disadvantages depending on the topology formation, the density of the IoT sensor network (which is equivalent to the average number of neighbors of a sensor node) and the number of storage nodes available. An important metric to be considered here is the minimum cluster population where the algorithms eliminate all conflicts, as well as the population of conflicts observed for lower populations of clusters. The fewer conflicts occur in the network, the better the protection of privacy achieved is.

Based on the analysis of the algorithms carried out, we have come to some conclusions. Initially, the pck-means algorithm performs, in almost all cases, better than the other two when the network consists of a small number of storage nodes, and mainly for *K* < 5. This is particularly important in terms of cost as the addition of new storage nodes will greatly increases the cost of the IoT infrastructure. However, because of the randomness it uses in initializing its centers, it demonstrates high instability for *K* > 4, rendering it unreliable as the overall number of storage nodes increases. For rather low density sensor networks, where a relatively low average number of sensor node neighbors is observed given the network size, the walkthrough algorithm convergences faster than the other two algorithms, achieving fewer conflicts in the majority of cases. However, for cases where the average population of sensor node neighbors increases leading to dense network topologies, the walkthrough algorithm is significantly outperformed by the other two algorithms. Moreover, its stability is questionable as the number of conflicts it generates can vary considerably between sequential executions of the algorithm for identical populations of storage nodes. The constrained agglomerative clustering algorithm does seem to demonstrate a particular advantage over the other two, but it is a stable algorithm that even when it is outperformed by the other two algorithms, the conflicts observed remain at acceptable levels.

Finally, in addition to the privacy implications introduced by high conflict populations, another metric that one may wish to evaluate the algorithms’ performance against is the large impact of the conflicts generated over the power consumption of the IoT sensor network. The lower the conflicts’ population is, the lower the population of sensor nodes assigned to cluster *m* + 1 is, and thus the lower the overall number of “messages of interest” exchanged between them, therefore reducing the power consumption required for posting such messages.

### 6.4. Dynamic Clustering Evaluation

**Figure 10:**
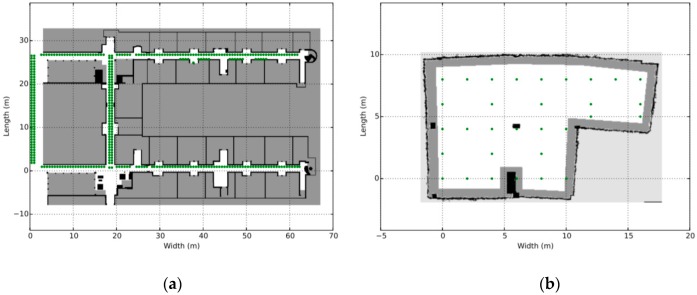
The construction of the location where the robots reside and the corridors where they can move [47] (**a**) Grenoble site, (**b**) Strasbourg site.

In this section, the evaluation results obtained by the experiments regarding the dynamic clustering are discussed. In this respect the performance of the DCA proposed is compared to the performance of the walkthrough algorithm, which appeared to be the most dominant so far for static clustering purposes. Several diagrams in this section present the level of privacy that each algorithm achieves over time, where the DCA results are depicted by the red lines, while the results of the walkthrough algorithm are depicted by the blue lines. The tables of this section present the average level of privacy that each algorithm achieves in the different experiments. Three trajectories for the mobile objects have been used that correspond to the ones specified and supported by FIT-IoT lab, i.e., trajectory 1 (mapped to the FIT-IoT square_1), trajectory 2 (mapped to the FIT-IoT h_line_2) and trajectory 3 (mapped to the FIT-IoT v_line_5). As stated in Section 6.1, the trajectories of the robots, which were used for the experiments, were selected by a predetermined pool of tracks as provided by FIT-IoT Lab. As illustrated in Figure 10, the locations of FIT-IoT Lab and the corridors, where the robot can move, are constructed quite symmetrically. The vast majority of these predetermined trajectories are square and linear tracks across the site. The most representatives of these tracks have been used in the conducted experiments.

#### 6.4.1. Experiments over 37 Node Topology

**Figure 11:**
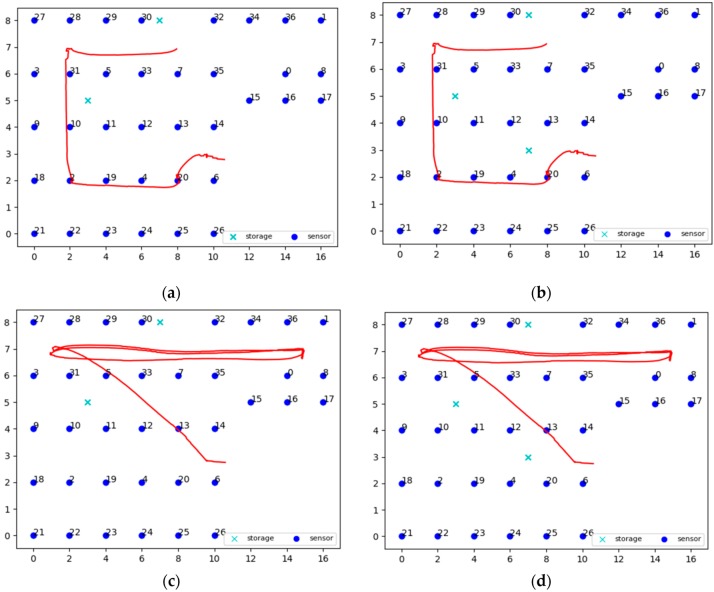
Trajectories over the topology of 37 nodes with *r_d_* = 1.5 and α = 2 for (**a**) 2 servers and square_1 trajectory, (**b**) 3 servers and square 1 trajectory, (**c**) 2 servers and h_line_2 trajectory, (**d**) 3 servers and h_line_2 trajectory.

**Figure 12:**
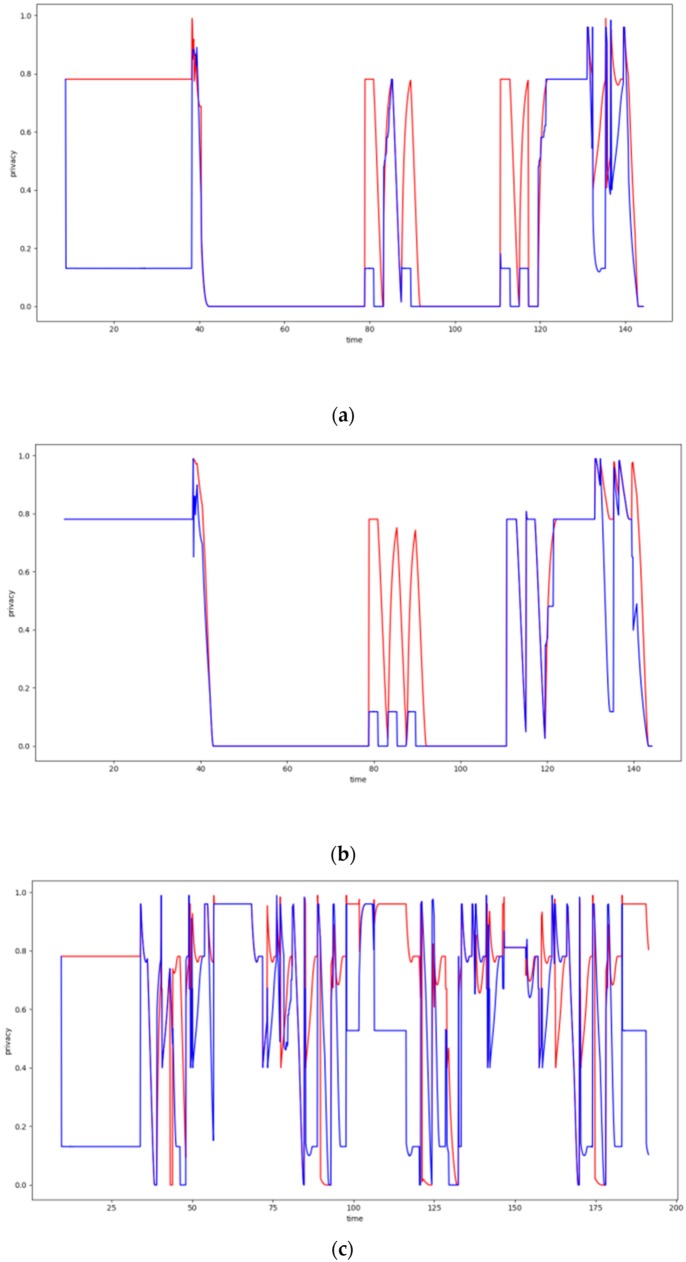

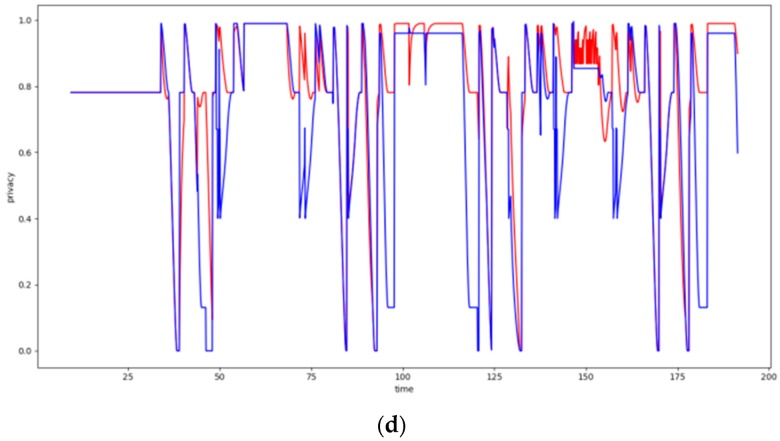
Evaluation results for dynamic clustering over the topology of 37 nodes for (**a**) 2 servers and square_1 trajectory, (**b**) 3 servers and square 1 trajectory, (**c**) 2 servers and h_line_2 trajectory, (**d**) 3 servers and h_line_2 trajectory.

In this section, the performance of the DCA is evaluated upon over a 37 node topology using two or three storage nodes and the trajectories depicted in Figure 11. The obtained evaluation results are presented in Figure 12. Moreover, Table 1 and Table 2 present the average privacy achieved by the two algorithms over the same experiments settings in the topology of 37 sensor nodes. Each row corresponds to a different population of storage nodes in the experiment, while each column corresponds to a different trajectory of the moving target. The respective results presented in Table 1 and Table 2 clearly indicate the advantages of the DCA over the walkthrough algorithm with respect to the average privacy ensured for all experiment settings conducted over the 37 sensor nodes topology. Overall, the DCA manages to increase the level of privacy ensured by the walkthrough algorithm by 33%, which is considerable. The less the storage nodes available are, the more significantly the walkthrough algorithm is outperformed by the DCA, which increases the average privacy it achieved up to 133%. It is important that even in the worst case scenario of only two storage nodes, dense sensing grid and quite distributed mobile object trajectory, the DCA manages to ensure average privacy of about 38%, while the respective performance of the walkthrough is only about 16%.

#### 6.4.2. Experiments over 100 Node Topology

**Figure 13:**
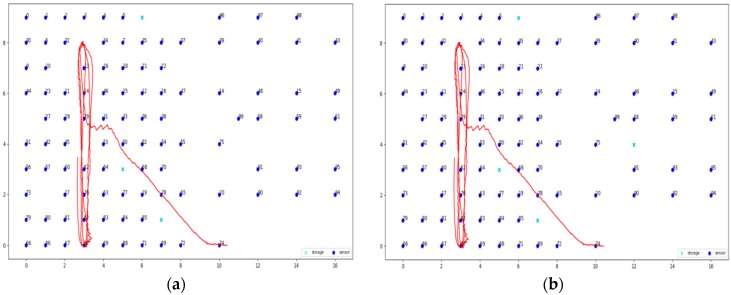

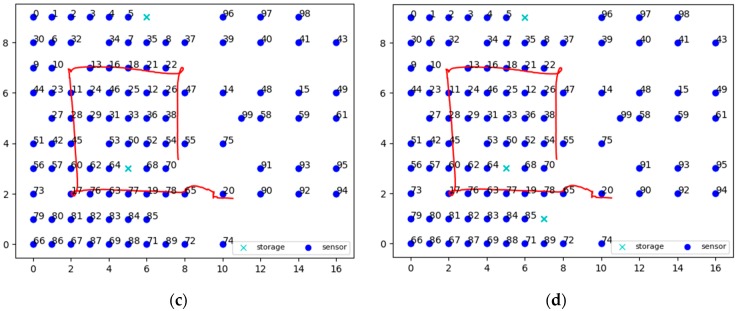
Trajectories over the topology of 100 nodes for (**a**) 3 servers, h_line_2 trajectory, *r_d_* = 4.5 *ans* α = 2.6, (**b**) 4 servers, h_line_2 trajectory, *r*_d_ = 4.5 *ans* α = 2.6, (**c**) 2 servers, square_1_plus trajectory, *r_d_* = 3 *ans* α = 0, (**d**) 3 servers, square_1_plus trajectory, *r_d_* = 3 *ans* α = 0.

**Figure 14:**
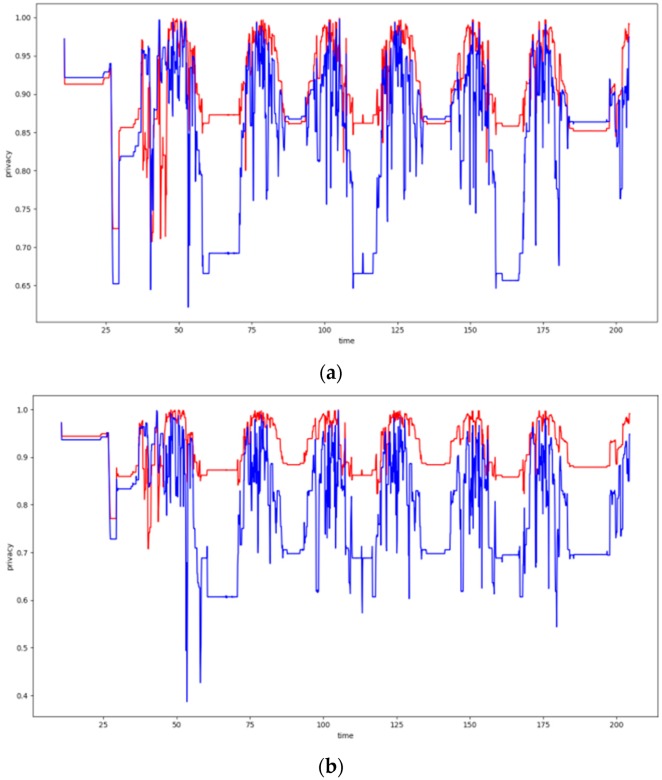

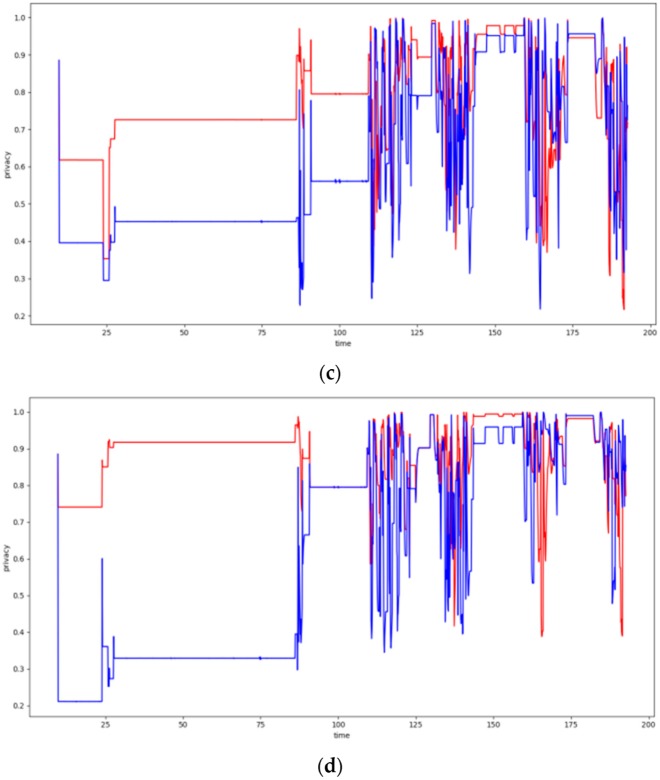
Evaluation results for dynamic clustering over the topology of 100 nodes for (**a**) 3 servers and square_1_plus trajectory, (**b**) 4 servers and square_1_plus trajectory, (**c**) 2 servers and h_line_2 trajectory, (**d**) 2 servers and h_line_2 trajectory.

In this section, the performance of the DCA is evaluated upon over a 100 node topology using two, three or four storage nodes and the trajectories depicted in Figure 13. The obtained evaluation results are presented in Figure 14. Moreover, Table 3 and Table 4 present the average privacy achieved by the two algorithms over the same experiments settings in the topology of 100 sensor nodes. Lack of specific figures in these tables indicates that no experiment has been conducted under the specific settings. The evaluation results presented in these tables clearly indicate that the DCA outperforms the walkthrough algorithm in all four series of experiments conducted over the 100 sensor nodes topology.

Overall, the DCA manages to increase the level of privacy ensured by the walkthrough algorithm by over 22%, as it ensures 87.3% average privacy versus 71.4% ensured by the walkthrough (averages across all 4 series of experiments). As previously observed, the fewer the storage nodes available are, the more significantly the DCA outperforms the walkthrough algorithm increasing its average privacy by up to 45%. Finally, it should be highlighted that over the experiments conducted for Square 1 trajectory of the mobile object, even though the walkthrough algorithm manages to eliminate all conflicts for two or more storage nodes available, it only ensures average privacy of about 60%. The DCA on the other hand, for the same settings delivers average privacy of 77% or 88%, respectively.

#### 6.4.3. Experiments over 123 Node Topology

**Figure 15:**
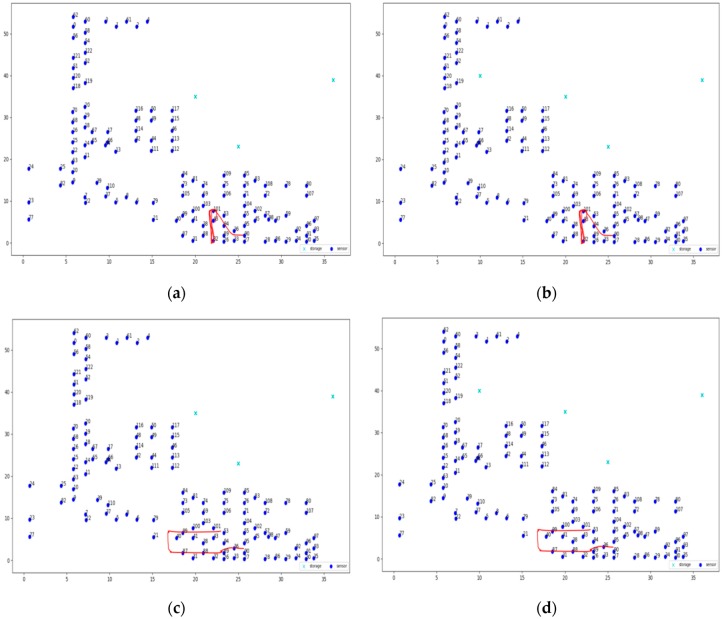
Trajectories over the topology of 123 nodes for (**a**) 3 servers and v_line_5 trajectory, (**b**) 4 servers and v_line_5, (**c**) 3 servers and square_1 trajectory, (**d**) 4 servers and square_1 trajectory.

**Figure 16:**
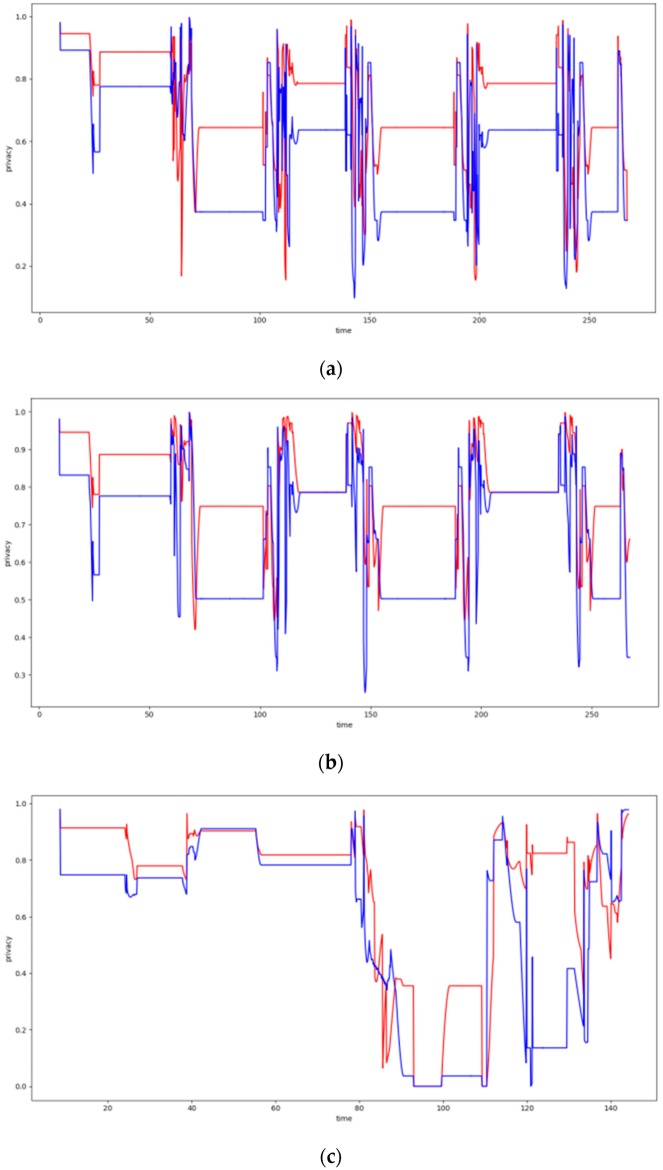

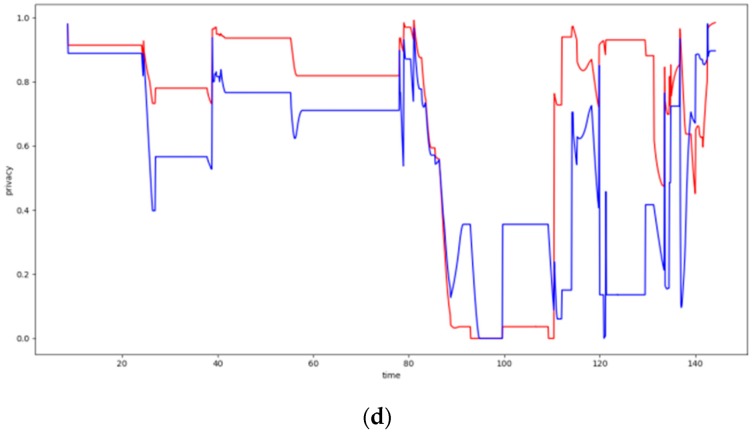
Evaluation results for dynamic clustering over the topology of 123 nodes for (**a**) 3 servers and v_line_5 trajectory, (**b**) 4 servers and v_line_5 trajectory, (**c**) 3 servers and square_1 trajectory, (**d**) 4 servers and square_1 trajectory.

In this section, the performance of the DCA is evaluated upon over a 123 node topology using three or four storage nodes and the trajectories depicted in Figure 15. The obtained evaluation results are presented in Figure 16. Moreover, Table 5 and Table 6 above present the average privacy achieved by the two algorithms over the same experiments settings in the topology of 123 sensor nodes. More specifically, four families of experiments have been conducted for 3 or 4 storage nodes and for square_1 or v_line_5 trajectories of the mobile object. The improvement in the average privacy achieved by DCA is evident, leading to an increase of the walkthrough average privacy (43.2%) of about 70% over the entire experiment suite executed, as the respective average privacy ensured by the DCA across all experiments is 73.4%.

Similar to the previous topologies, the fewer the storage nodes available are, the more considerable the DCA outperforms the walkthrough algorithm, increasing its average privacy up to 28%. It should also be highlighted that the average privacy ensured by the DCA is always 70%–80%, which is quite appropriate for such dense networks and few storage nodes.

#### 6.4.4. Overall Evaluation Results

Over all experiments conducted, the proposed DCA outperforms the walkthrough algorithm ensuring higher average privacy in all circumstances. Based on the evaluation findings formerly discussed, the DCA outperforms the walkthrough algorithm for all mobile trajectories, detection ranges, sensor node populations and network topology densities tested. A very important observation is that the way nodes are selected to dynamically change their cluster has a great effect on the efficiency of the algorithm. Based on experience and experimental results, the selective method proved to be the most appropriate because the nodes selected are more scattered over the network and not concentrated to a specific part of the network. This is easier to manage as no neighborhoods with a large number of nodes that dynamically change their cluster are created, leading to heavier synchronization problems, power consumption and computational power needs as messages are constantly exchanged between them.

The choice of the method used in the first step of the DCA is critical to the outcome it achieved. The randomness of the walkthrough algorithm can lead to unexpected changes over the privacy metric values, and consequently to the average privacy ensured. While the DCA controls this randomness and leads to an improved result, it has nevertheless been preferred to use constrained agglomerative clustering, which in some cases may create more conflicts than the walkthrough, yet they are kept at acceptable levels. In addition, the conflicts created with this algorithm are consistent in every successive execution, enabling the DCA to ensure a stable level of privacy.

As can be seen from the figures above, there are some limited intervals during the experiments’ execution, where the walkthrough algorithm delivers higher privacy than the DCA. This is due to the fact that problems in the privacy level of the network may also be caused by nodes located in distance less than 2*r_d_* − *a*, for which the privacy problems cannot be eliminated. The two algorithms can cluster these nodes in a different way and in some cases the clustering of the walkthrough is more efficient. Especially if the DCA does not assign to one of them the non-existent cluster *m* + 1, this clustering will remain unchanged until the end of the experiment. However, in the proposed algorithm, this improvement can be done at the preprocessing stage so that this problem is managed in node neighborhoods that are very close to each other and have been clustered in a way that creates problematic issues after the completion of the first cluster layer. However, even without this improvement, the DCA achieves better privacy than the walkthrough in the overwhelming part of the duration of all experiments.

Finally, as expected the DCA achieves higher privacy as the number of storage nodes increases. This is reasonable if we take into consideration that a node, which changes its group in a dynamic way, is able to choose from a larger population of storage nodes. On the other hand, walkthrough is not a stable algorithm regarding the increase of storage nodes. This instability is caused by the fact that walkthrough includes random functions that may initially formulate fully inappropriate sensor nodes clusters. However, in DCA the sensor nodes, which in time *t* cannot be assigned to an available cluster, select to be assigned to the least used storage node. This criterion resolves the respective problem imposed by the walkthrough algorithm. After the static and dynamic analysis of the algorithms, it is evident that DCA leads to better privacy level regardless of the conditions of the topology or the trajectory of the mobile node.

## 7. Conclusions

In the context of this article, a new data transmission protocol has been designed and implemented in order to protect sensitive location data from aspiring intruders. The proposed techniques do not focus on the prevention of malicious attacks, but on enhancing the privacy of sensitive location information once IoT nodes have been compromised. In this respect, a new two-level constrained clustering algorithm is introduced that improves the privacy ensured across IoT environments. This is the Dynamic Clustering Algorithm (DCA) that specifies three phases: the initial one corresponds to the employment of existing algorithms that deliver an initial sensor node clustering and to the selection of the sensor nodes to be attached to an extra cluster (not assigned to a specific storage node), the second phase that aims to further process and improve this initial clustering and the third phase manages the nodes on the fly, dynamically reformulating the sensor node clusters based on the current location of the mobile object. Some of the most common and efficient constrained clustering algorithms such as pck-means, constrained agglomerative clustering and walkthrough have been used for the first step of DCA and the static network clustering, while the rest of the DCA has been specified so that the number of conflicts induced by sensor nodes that co-detect the mobile object at the same time, while being assigned to the same storage node is minimised.

To evaluate the performance of the DCA, series of experiments have been conducted over the IoT infrastructure of FIT IoT-LAB based on various topologies and trajectories of mobile object. Thus, no simulations have been used for the DCA assessment purposes, as all experiments have been executed over IoT testbeds deployed by FIT-IoT Lab. The evaluation results obtained indicate that the level of privacy that can be achieved by the DCA is always higher (often significantly) than that of conventional constrained clustering algorithms regardless of the experiment settings and respective configurations, i.e., for varying sensor node populations, storage node populations, sensor network density and topology formations, sensing range and mobile object trajectories. Thus, it is concluded that the dynamic clustering algorithm proposed considerably outperforms existing solutions focusing on enhancing the privacy of location information in IoT, increasing the respective performance of the most suitable clustering algorithm by up to 133%.

The authors plan to proceed with the research work presented in this article. Their future plans include the following: evaluating the scalability of the DCA testing its performance over much larger topologies of thousands of sensor nodes; evaluate the impact of the DCA on sensor node power consumption especially when multiple mobile targets enter the network; and design an enhanced DCA that will jointly increase the average privacy protection ensured and reduce the respective power consumption induced. Finally, as it is not straight forward to find a reliable and fully secure Global node in real-world scenarios, it lies among the authors plans to adapt and evaluate the designed mechanisms in a setting where the Global node is replaced by a secure mobile node that will periodically and selectively aggregate the location information originally collected by the storage nodes of the IoT infrastructure.

## Figures and Tables

**Table 1:** Average privacy achieved by the walkthrough algorithm for the topology of 37 nodes.

Average Privacy	square_1	h_line_2
2 storage nodes	0.1643	0.5108
3 storage nodes	0.3489	0.7018

**Table 2:** Average privacy achieved by the DCA for the topology of 37 nodes.

Average Privacy	square_1	h_line_2
2 storage nodes	0.3830	0.7180
3 storage nodes	0.4108	0.7898

**Table 3:** Average privacy achieved by the walkthrough algorithm for the topology of 100 nodes.

Average Privacy	square_1_plus	h_line_2
2 storage nodes	-	0.6088
3 storage nodes	0.8477	0.6084
4 storage nodes	0.7917	-

**Table 4:** Average privacy achieved by the DCA for the topology of 100 nodes.

Average Privacy	square_1_plus	h_line_2
2 storage nodes	-	0.7707
3 storage nodes	0.9135	0.8817
4 storage nodes	0.9248	-

**Table 5:** Average privacy achieved by the walkthrough algorithm for the topology of 123 nodes.

Average Privacy	square_1	v_line_5
3 storage nodes	0.5608	0.5708
4 storage nodes	0.5978	0. 6877

**Table 6:** Average privacy achieved by the DCA for the topology of 123 nodes.

Average Privacy	square_1	v_line_5
3 storage nodes	0.7039	0.7289
4 storage nodes	0.7046	0.7989

## Appendix A

*Walkthrough*: It constitutes a distributed clustering algorithm that is applied to each and every node of the network aiming at partitioning them into groups under certain distance constraints. For this reason, it employs a Spatial Privacy Graph [2] that connects with an edge the pairs of sensor nodes (*xi*, *xj*) whose distance belong within a certain distance interval. The algorithm starts with the nodes assigned in different clusters whose number is greater than the expected. The objective of the algorithm is to reduce the number of clusters by assigning the nodes in valid and feasible clusters based on the one that their neighbors in SPG belong.

*Constrained Hierarchical Agglomerative*: It constitutes a hierarchical clustering algorithm [39,40] that belongs in the family of semi-supervised clustering algorithms. It utilizes data that have known and predetermined constraints that can be classified into two categories. The first is “must-link” constraints which consist of pair of data that have to be grouped into the same cluster and the second is “cannot-link” constraints which include observations that have to be classified into different clusters. It starts with each observation classified into different cluster and performs an aggregation of clusters in each step. If a pair of data is affected by a “must-link” then the distance of the corresponding clusters is set to 0. In the opposite case that the pair of data belongs to “cannot-link” constraints, the distance is set to the maximum possible distance value.

*PCk-means*: It constitutes an expansion of traditional kmeans algorithm [39,40] to include predetermined constraints between the observations. It takes as input a set of “must-link” and “cannot-link” constraints that have to respect. Its first stage is to take the transitive closure of these constraints and initialize the centroids of the clusters based on the centroids of the largest neighborhoods or random perturbations of the global centroid of the data. For each constraint that does not respect adds a penalty to an objective function. Its aim is to minimize this objective function so as to violate the least possible number of constraints.
